# HGF-mediated elevation of ETV1 facilitates hepatocellular carcinoma metastasis through upregulating PTK2 and c-MET

**DOI:** 10.1186/s13046-022-02475-2

**Published:** 2022-09-16

**Authors:** Tongyue Zhang, Yijun Wang, Meng Xie, Xiaoyu Ji, Xiangyuan Luo, Xiaoping Chen, Bixiang Zhang, Danfei Liu, Yangyang Feng, Mengyu Sun, Wenjie Huang, Limin Xia

**Affiliations:** 1 Department of Gastroenterology, Institute of Liver and Gastrointestinal Diseases, Hubei Key Laboratory of Hepato-Pancreato-Biliary Diseases, Tongji Hospital of Tongji Medical College, Huazhong University of Science and Technology, Wuhan, 430030 Hubei Province China; 2 Hepatic Surgery Center, Hubei Key Laboratory of Hepato-Pancreato-Biliary Diseases, Tongji Hospital of Tongji Medical College, Huazhong University of Science and Technology, Wuhan, 430030 Hubei Province China; 3 Key Laboratory of Organ Transplantation, Ministry of Education and Ministry of Public Health, Wuhan, 430030 Hubei China

**Keywords:** E-twenty-six transformation-specific variant 1, Tyrosine protein kinase Met, Protein tyrosine kinase 2, Defactinib, Capmatinib

## Abstract

**Background:**

Metastasis is a major determinant of death in patients with hepatocellular carcinoma (HCC). Dissecting key molecular mediators that promote this malignant feature may help yield novel therapeutic insights. Here, we investigated the role of E-twenty-six transformation-specific variant 1 (ETV1), a member of the E-twenty-six transformation-specific (ETS) family, in HCC metastasis.

**Methods:**

The clinical significance of ETV1 and its target genes in two independent cohorts of HCC patients who underwent curative resection were assessed by Kaplan–Meier analysis and Multivariate Cox proportional hazards model. Luciferase reporter assay and chromatin immunoprecipitation assay were used to detect the transcriptional regulation of target gene promoters by ETV1. The effect of ETV1 on invasiveness and metastasis of HCC were detected by transwell assays and the orthotopically metastatic model.

**Results:**

ETV1 expression was frequently elevated in human HCC specimens. Increased ETV1 expression was associated with the malignant biological characteristics and poor prognosis of HCC patients. ETV1 facilitated invasion and metastasis of HCC cells in vitro and in vivo. Mechanistically, ETV1 promoted HCC metastasis via upregulating metastasis-related genes, including *protein tyrosine kinase 2* (*PTK2*) and *MET.* Down-regulated the expression of PTK2 or tyrosine protein kinase Met (c-MET) decreased ETV1-mediated HCC metastasis. Hepatocyte growth factor (HGF) upregulated ETV1 expression through activating c-MET-ERK1/2-ELK1 pathway. Notably, in two independent cohorts, patients with positive coexpression of ETV1/PTK2 or ETV1/c-MET had worse prognosis. Furthermore, the combination of PTK2 inhibitor defactinib and c-MET inhibitor capmatinib significantly suppressed HCC metastasis induced by ETV1.

**Conclusion:**

This study uncovers functional and prognostic roles for ETV1 in HCC and exposes a positive feedback loop of HGF-ERK1/2-ETV1-c-MET. Targeting this pathway may provide a potential therapeutic intervention for ETV1-overexpressing HCC.

**Supplementary Information:**

The online version contains supplementary material available at 10.1186/s13046-022-02475-2.

## Background

Hepatocellular carcinoma (HCC) ranks the third leading cause of cancer-related deaths around the world [1]. Metastasis and recurrence often lead to the deaths of HCC patients [2, 3]. Despite the diagnostic methods and treatments have been a step forward after decades of efforts, only a minority of unselected patients benefit [4]. Due to the complex molecular pathogenesis and the lack of appropriate biomarkers, many targeted drugs used as a single agent, such as sorafenib and tivantinib, did not produce ideal clinical benefits [5–7]. To date, the five-year survival rate of advanced HCC is still low [7]. For this reason, attention has been drawn to new combination strategies for better therapeutic efficacy. For example, bevacizumab plus atezolizumab was approved as the first-line treatment for advanced HCC patients for bringing better survival benefits than sorafenib [8–10]. At present, substantial clinical trials involving combination therapy are in progress. Accordingly, it is essential to gain a more thorough comprehension of the molecular mechanism responsible for HCC metastasis and propose potential therapies that are most likely to benefit a certain patient population.

The human E-twenty-six transformation-specific (ETS) family comprises a group of evolutionarily conserved transcription factors, characterized by a highly conserved ETS domain in the C-terminal region recognizing 5’-GGAA/T-3’ motif. It has 28 members belonging to 12 separate clusters [11]. ETS factors govern a plethora of developmental and physiological processes, including cell proliferation, differentiation, migration, tissue remodeling, and angiogenesis [11]. ETV1, also named ETS-related protein 81 (ER81), is a member of the polyomavirus enhancer activator 3 (PEA3) subfamily(ETV1, ETV4, ETV5), which is featured with an acidic transactivation domain at the N-terminus [12]. In development, ETV1 performs physiological functions in branching morphogenesis, rapid conduction physiology in the heart and motor coordination [13–15]. Knockout of ETV1 in mice usually leads to motor discoordination and death in the third weeks of birth [15, 16]. ETV1 dysregulation leads to initiation and progression of multiple types of tumors. For example, ETV1 is frequently dysregulated in prostate cancer and reported to be overexpressed in the most aggressive prostate tumors [17]. ETV1 induces cell migration and invasion of prostate cancer via stabilizing β-catenin and directly binding to MMP1/7 [18, 19]. In pancreatic ductal adenocarcinoma, ETV1 expression is increased in primary and metastatic specimens. It facilitates epithelial-mesenchymal transition (EMT), stromal expansion and metastasis through regulating hyaluronic acid synthase 2 (HAS2) and secreted protein acidic and rich in cysteine (SPARC) in mice [20]. In addition, ETV1 is also known to be required for gastrointestinal stromal tumor initiation and proliferation. It plays as a master regulator in oncogenic transcriptional programme and forms a feedback circuit together with mutant KIT [21]. Nevertheless, the expression and the functional role of ETV1 in HCC remain elusive.

Accumulating data indicate a central role for the hepatocyte growth factor/tyrosine protein kinase Met (HGF/c‐Met) pathway in HCC metastasis. Physiologically, the HGF/c-MET axis participates in embryogenesis, cell growth and movement, tissue morphogenesis, morphological differentiation, and organ regeneration [22]. Upon binding to HGF, dimerization and trans-autophosphorylation of c-MET activate various downstream signaling pathways, including the ERK1/2/JNKs/p38 MAPK and the PI3K-Akt pathway, which reach the nucleus to affect gene expression involving angiogenesis and metastasis [22–24]. In HCC, HGF promotes EMT and enhances invasion of HCC cells with increased expression of MMP9 and Snail [25, 26]. In human HCC samples, c-MET expression is associated with intrahepatic metastatic nodules and vascular invasion [27–29]. Patients with HCC and high expression of c-MET were reported to have an early recurrence and poor survival time [28, 30]. Thus, c-MET is considered an intriguing therapeutic target for HCC. However, no selective c-MET inhibitors have been approved for HCC patients, which calls for further study.

This study revealed the clinicopathological significance and function of ETV1 in HCC. Elevated ETV1 expression indicated poor prognosis in HCC and promoted HCC metastasis via upregulating PTK2 and c-MET. HGF/c-MET signaling enhanced ETV1 expression through ERK1/2 activation, which in turn upregulated c-MET expression, forming a positive feedback circuit. Combined treatment of PTK2 inhibitor plus c-MET inhibitor impeded ETV1-induced HCC metastasis.

## Methods

### Experimental metastasis mouse model

Male 5-week-old BALB/C nude mice were raised under standard conditions. Animal experiments were approved by the Committee on the Tongji Hospital of Tongji Medical College, Huazhong University of Science and Technology. In the orthotopic xenograft model, human fluorescein-labeled 2.0 × 10^6^ HCC cells were resuspended at 50 μl phosphate-buffered saline and mixed with 50 μl matrige injected into the left lobe livers of nude mice (ten per group). Defactinib was orally administered at a dose of 25 mg/kg twice a day, capmatinib was orally administered at a dose of 10 mg/kg/day. The formation of the tumors were detected by bioluminescence. D-luciferin (Perkin-Elmer) was intraperitoneally injected into the mice to detect signals. The bioluminescence images were obtained by the Lago X optical imaging system (SI Imaging). At the predetermined endpoint (9 weeks), the livers and lungs of nude mice were preserved for histological study.

### The human liver cancer RT^2^ profiler PCR array

The Human Liver Cancer RT^2^Profiler PCR Array (Cat. no. 330231 PAHS-133ZA)profiles the expression of 84 key genes involved in the progression of hepatocellular carcinoma (HCC), as well as other forms of hepatocarcinogenesis. This array includes genes commonly up-and down-regulated in HCC, genes involved in commonly altered signal transduction pathways, and genes involved in other dysregulated biological pathways such as epithelial to mesenchymal transition, cell adhesion, apoptosis, and inflammation. PLC/PRF/5-control and PLC/PRF/5-ETV1, MHCC97H-shControl cells and MHCC97H-shETV1 cells were used. RNA extraction, DNase treatment, and RNA cleanup were performed according to the manufacturer’s protocol (Qiagen). The cDNA of each group was synthesized using the RT2 First Strand Kit (Qiagen). Gene expression profiling of each group was conducted using the Human liver cancer RT2 Profiler PCR Array, stored at -20℃. The cDNA synthesis reaction was mixed with 2 × RT^2^ qPCR SYBR Green Mastermix and ddH2O, and then dispensed to the PCR array 96-well plate (25 μL/well). A 2-step cycling program was performed using the Bio-Rad CFX96. Data normalization was done by correcting all Ct values based on the average Ct values of several housekeeping genes present on array. Each assay was conducted in triplicate.

### Immunohistochemistry

First, 4-μm-thick paraffin tissue sections were baked at 60 °C for one hour to deparaffinize and treated with 3% hydrogen peroxide in methanol. After 15 min, the tissue sections were washed with phosphate-buffered saline. Then the microwave antigen repair was used. Next, sections were incubated overnight at 4 °C with the primary antibody. The tissue microarrays were stained for ETV1 (Ruiying, RLT1604), PTK2 (Cell Signaling Technology, #3285), and c-MET (Cell Signaling Technology, #8198). The use of pre-immunized mouse serum was used as the negative control. Slides were incubated with the peroxidase-conjugated second antibody (Santa Cruz) for half an hour and then visualized with diaminobenzidine. Finally, the images were acquired through a light microscope (Olympus, Japan) with a DP70 digital camera.

### Agents

PI3K inhibitor LY294002, ERK1/2 inhibitor SCH772984, JNK inhibitor SP600125, p38 inhibitor SB203580, capmatinib and defactinib were purchased from MedChemExpress. The agents were used under the standard protocols. The HCC cells were pretreated with PI3K inhibitor LY294002 (10 μM), ERK1/2 inhibitor SCH772984 (10 μM), JNK inhibitor SP600125 (10 μM), or p38 inhibitor SB203580 (10 μM) for 1 h. Defactinib was orally administered at a dose of 25 mg/kg twice a day, capmatinib was orally administered at a dose of 10 mg/kg/day. The treatment began on the seventh day after the establishment of the animal model and lasted eight weeks.

### Statistical analysis

All data were displayed as the mean ± standard deviation (SD). Student’s t-tests, Mann–Whitney U tests, and Wilcoxon signed-rank test were used for comparison between data of two groups with normal distribution, abnormal distribution, and matched pairs, respectively. One-way ANOVA, followed by Tukey’s post hoc analysis, was used for comparison among multiple groups. χ2 test was for categorical data. Kaplan–Meier analysis (log-rank test) was applied to depict the cumulative recurrence rates and overall survival curves. Multivariate analysis was carried out through Cox regression analysis. The SPSS 20.0 software and GraphPad Prism 8.0 were employed for statistical analyses. A value of *p* < 0.05 was considered to be significant.

The exhaustive information of materials and methods were described in the supplementary materials and methods.

## Results

### Highly-expressed ETV1 indicates poor prognosis in HCC and displays a promoting effect on HCC metastasis

**Fig. 1:**
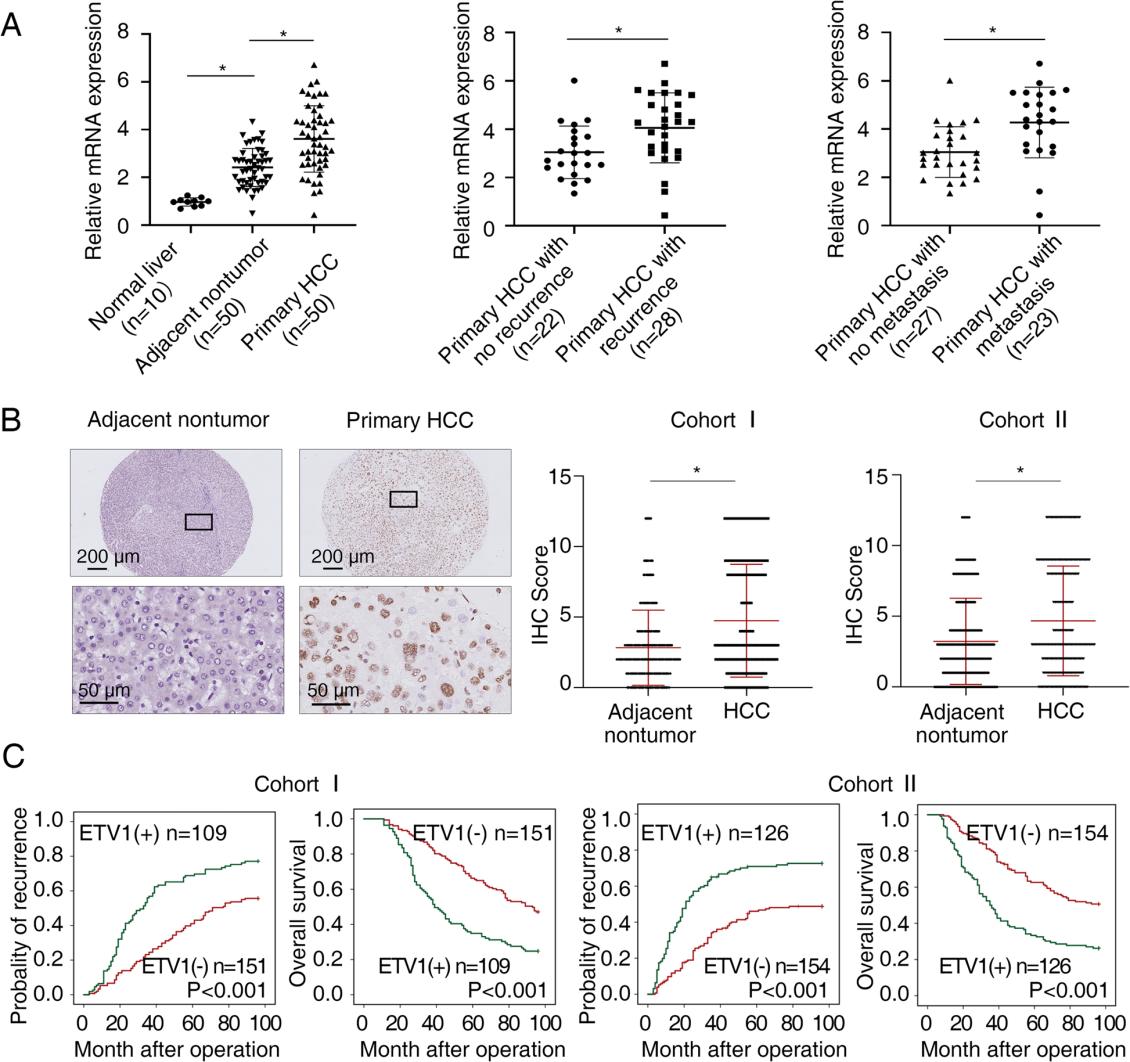
Highly-expressed ETV1 indicates poor prognosis in HCC. (**A**) The relative ETV1 mRNA level in 10 cases of normal liver specimens, as well as 50 cases of primary HCC specimens and paired paracancerous nontumorous specimens was detected by RT-qPCR (left). ETV1 mRNA levels in 22 cases of primary HCC specimens without recurrence and 28 cases with recurrence (middle). ETV1 mRNA levels in 27 cases of primary HCC specimens without metastasis and 23 cases with metastasis (right). (**B**) Representative IHC staining (left) and IHC scores (right) of ETV1 in paired primary HCC specimens and nontumorous specimens in two independent HCC cohorts. Scale bars, 200 µm (upper), 50 µm (lower). (**C**) OS and recurrence rates curves by Kaplan-Meier for HCC tissues with positive and negative ETV1 levels in two independent HCC cohorts. * Represented p < 0.05. All data were displayed as Mean ± SD

We tested the mRNA level of ETV1 by quantitative RT-PCR (RT-qPCR) in 10 normal liver specimens, as well as 50 matched pairs of adjacent nontumorous and primary HCC specimens. ETV1 was lowly expressed in human normal liver specimens, which is consistent with a previous report [31]. The mRNA level of ETV1 in primary HCC specimens was higher than that in adjacent nontumorous specimens or normal liver specimens (Fig. 1A left). In addition, as shown in the middle and right parts of Fig. 1A, the ETV1 expression in recurrent or metastatic HCC specimens was higher than those without recurrence or metastasis. Subsequently, immunohistochemical (IHC) staining was performed to detect ETV1 expression in two independent cohorts (cohort I, *n* = 260; cohort II, *n* = 280) (Fig. 1B). Consistent with the mRNA level, ETV1 protein level in human HCC specimens was higher when compared to paracancerous nontumorous specimens. Particularly, in both cohorts, increased ETV1 expression was significantly correlated with loss of tumor encapsulation, poor tumor differentiation, microvascular invasion, as well as advanced tumor nodular metastasis (TNM) stage (Table 1). As Kaplan–Meier survival analysis illustrated, the high ETV1 level in HCC patients was associated with higher recurrence risks and worse overall survival (OS) compared to low ETV1 expression (Fig. 1C). In concordance with our data, ETV1 was upregulated in tumor tissues, and the high ETV1 mRNA level was correlated with poor survival for patients with HCC in TCGA (Fig. S1A). Notably, multivariate analysis showed that the ETV1 level was an independent risk factor of recurrence and survival in HCC patients (Table 2). Altogether, our data demonstrated that increased ETV1 expression contributed to malignant characteristics of HCC and was associated with a high risk of recurrence and poor survival in HCC patients.

**Table 1:** Correlation between ETV1 expression and clinicopathological characteristics of HCCs in two independent cohorts of human HCC tissues

Clinicopathological variables		Tumor ETV1 expression		*P* Value	Tumor ETV1 expression		*P* Value
		Negative (*n* = 151)	Positive (*n* = 109)		Negative (*n* = 154)	Positive (*n* = 126)	
Age		52.11	51.77	0.411	50.57	51.51	0.351
		(11.915)	(10.417)		(10.053)	(9.743)	
Sex	female	31	18	0.428	19	25	0.100
	male	120	91		135	101	
Serum AFP	≤ 20 ng/ml	46	21	0.045	32	23	0.652
	> 20 ng/ml	105	88		122	103	
Virus infection	HBV	108	84	0.523	119	106	0.406
	HCV	14	11		11	8	
	HBV + HCV	9	3		11	4	
	none	20	11		13	8	
Cirrrhosis	absent	40	32	0.674	42	34	1.000
	present	111	77		112	92	
Child–pugh score	Class A	113	83	0.884	130	103	0.630
	Class B	38	26		24	23	
Tumor number	single	91	63	0.703	124	86	0.026
	multiple	60	46		30	40	
Maximal tumor size	≤ 5 cm	69	39	0.126	102	56	< 0.001
	> 5 cm	82	70		52	70	
Tumor encapsulation	present	95	50	0.008	118	77	0.006
	absent	56	59		36	49	
Microvascular invasion	absent	97	45	< 0.001	97	56	0.003
	present	54	64		57	70	
Tumor differentiation	I-II	131	75	0.001	132	77	< 0.001
	III-IV	20	34		22	49	
TNM stage	I-II	128	70	< 0.001	136	82	< 0.001
	III	23	39		18	44

**Table 2:** Univariate and multivariate analysis of factors associated with time to recurrence and overall survival in two independent cohorts of human HCC

**Cohort I (*N* = 260)**	Time To Recurrence		Overall Survival	
Clinical Variables	HR(95%CI)	*P* value	HR(95%CI)	*P* value
**Univariate Analysis**				
Age	0.982(0.969–0.995)	0.006	0.981(0.968–0.994)	0.005
Sex (male versus female)	0.925(0.629–1.360)	0.692	0.873(0.593–1.286)	0.492
Serum AFP (> 20 versus ≤ 20 ng/ml)	1.349(0.943–1.931)	0.102	1.405(0.970–2.036)	0.072
HBV infection (yes versus no)	0.792(0.564–1.112)	0.178	0.767(0.543–1.081)	0.130
Cirrhosis ( present versus absent)	0.903(0.647–1.259)	0.548	0.876(0.625–1.228)	0.442
Child–pugh score (B versus A)	0.959(0.675–1.364)	0.818	0.948(0.661–1.358)	0.769
Tumor number (multiple versus single)	1.961(1.447–2.656)	< 0.001	2.048(1.503–2.791)	< 0.001
Maximal tumor size (> 5 versus ≤ 5 cm)	1.510(1.103–2.066)	0.010	1.573(1.139–2.173)	0.006
Tumor encapsulation (absent versus present)	3.422(2.507–4.669)	< 0.001	3.584(2.607–4.927)	< 0.001
Microvascular invasion (present versus absent)	3.111(2.280–4.245)	< 0.001	3.315(2.411–4.558)	< 0.001
Tumor differentiation (III- IV versus I-II)	3.155(2.243–4.437)	< 0.001	3.363(2.385–4.743)	< 0.001
TNM stage (III versus I-II)	6.680(4.741–9.414)	< 0.001	7.093(5.015–10.033)	< 0.001
ETV1 (positive versus negative)	2.059(1.519–2.792)	< 0.001	2.143(1.571–2.922)	< 0.001
**Multivariate analysis**				
Tumor number (multiple versus single)	1.441(0.925–2.247)	0.106	1.530(0.973–2.406)	0.066
Maximal tumor size (> 5 versus ≤ 5 cm)	1.417(0.966–2.078)	0.075	1.465(0.986–2.175)	0.059
Tumor encapsulation (absent versus present)	1.335(0.840–2.121)	0.222	1.289(0.803–2.072)	0.293
Microvascular invasion (present versus absent)	1.663(1.082–2.556)	0.020	1.808(1.168–2.800)	0.008
Tumor differentiation (III- IV versus I-II)	1.569(1.075–2.288)	0.019	1.651(1.131–2.411)	0.009
TNM stage (III versus I-II)	3.004(1.815–4.971)	< 0.001	3.004(1.796–5.026)	< 0.001
ETV1 (positive versus negative)	1.651(1.201–2.269)	0.002	1.748(1.266–2.415)	0.001
**Univariate Analysis**				
Age	0.992(0.977–1.008)	0.327	0.988(0.972–1.003)	0.117
Sex (male versus female)	1.080(0.695–1.678)	0.732	0.987(0.645–1.510)	0.952
Serum AFP (> 20 versus ≤ 20 ng/ml)	0.951(0.657–1.377)	0.789	0.907(0.631–1.302)	0.595
HBV infection (yes versus no)	2.203(1.380–3.519)	0.001	2.305(1.444–3.679)	< 0.001
Cirrhosis ( present versus absent)	1.212(0.856–1.716)	0.278	1.242(0.878–1.756)	0.221
Child–pugh score (B versus A)	1.359(0.924–1.999)	0.120	1.339(0.911–1.969)	0.137
Tumor number (multiple versus single)	2.397(1.726–3.329)	< 0.001	2.391(1.724–3.316)	< 0.001
Maximal tumor size (> 5 versus ≤ 5 cm)	2.511(1.839–3.429)	< 0.001	2.317(1.705–3.151)	< 0.001
Tumor encapsulation (absent versus present)	3.058(2.239–4.177)	< 0.001	2.897(2.127–3.946)	< 0.001
Microvascular invasion (present versus absent)	2.213(1.624–3.016)	< 0.001	2.268(1.668–3.083)	< 0.001
Tumor differentiation (III- IV versus I-II)	3.539(2.561–4.890)	< 0.001	3.444(2.503–4.739)	< 0.001
TNM stage (III versus I-II)	5.481(3.913–7.675)	< 0.001	5.875(4.196–8.225)	< 0.001
ETV1 (positive versus negative)	2.218(1.629–3.020)	< 0.001	2.220(1.635–3.014)	< 0.001
**Multivariate analysis**				
Tumor number (multiple versus single)	1.272(0.852–1.898)	0.239	1.236(0.823–1.857)	0.307
Maximal tumor size (> 5 versus ≤ 5 cm)	1.146(0.790–1.662)	0.473	1.013(0.704–1.456)	0.946
Tumor encapsulation (absent versus present)	1.495(0.986–2.269)	0.059	1.394(0.923–2.105)	0.114
Microvascular invasion (present versus absent)	1.475(0.995–2.188)	0.053	1.625(1.101–2.399)	0.015
Tumor differentiation (III- IV versus I-II)	1.727(1.195–2.495)	0.004	1.696(1.180–2.437)	0.004
TNM stage (III versus I-II)	2.968(1.885–4.672)	< 0.001	3.518(2.230–5.550)	< 0.001
ETV1 (positive versus negative)	1.636(1.173–2.283)	0.004	1.638(1.180–2.274)	0.003

**Fig. 2:**
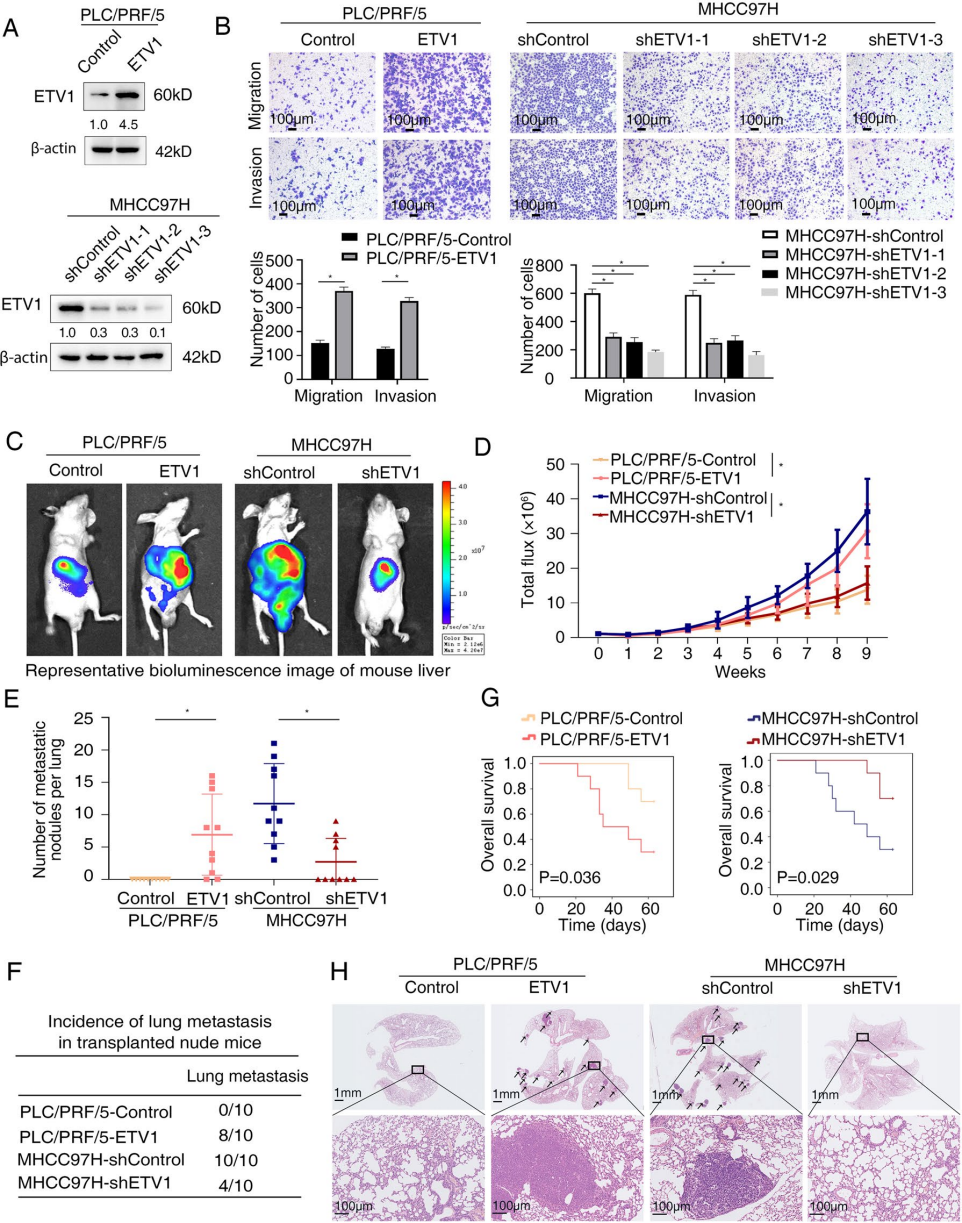
ETV1 promotes HCC metastasis in vitro and in vivo. (**A**) The efficiency of ETV1-overexpression and ETV1-knockdown in the indicated HCC cells was detected by western blotting. (**B**) Effects of ETV1 overexpression and knockdown on HCC cell migration and invasion. Scale bar, 100 μm. (**C**-**H**) In vivo metastatic assay. (**C**) The representative BLI images in the liver were shown nine weeks after implanting with indicated cells. (**D**) The bioluminescent signals were used to show the growth rate of liver tumors. (**E**) The number of metastatic lesions in the lung tissues. (**F**) The occurrence of lung metastasis. (**G**) The OS of different groups in the in vivo models. (**H**) Representative images of H&E staining of lung samples (indicated by arrowheads) from each group. Scale bars, 1mm(upper), 100μm (lower). * Represented p < 0.05. All data were displayed as Mean ± SD

To interrogate the function of ETV1 in HCC, we first assessed the mRNA and protein levels in a panel of HCC cell lines. High endogenous ETV1 expression was confirmed in multiple HCC cells, especially in those with high metastatic potential (HCCLM3, HCCLM6, and MHCC97H), which suggested its potential role in HCC metastasis (Fig. S1 B-C). We then generated stable PLC/PRF/5-ETV1 cell line and MHCC97H-shETV1 cell line (Fig. 2A). As shown in Fig. 2B, cell migration and invasion, detected by transwell assays, were increased in PLC/PRF/5-ETV1 cells and decreased in MHCC97H-shETV1 cells when compared to their controls. Based on the knockdown effect, the shETV1-3 was chosen for the following studies. Furthermore, cell proliferation, as measured by CCK-8 assay and colony formation assay, was enhanced upon overexpression of ETV1 and vice versa (Fig. S1 D-E).

Next, we examined whether ETV1 is responsible for tumor growth and metastasis in vivo. We performed an orthotopically metastatic model and measured with the bioluminescence imaging (BLI) signals. Compared with PLC/PRF/5-Control group, PLC/PRF/5-ETV1 group demonstrated increased bioluminescence intensity, lung metastasis incidence, and lung metastatic nodules number, together with shortened OS. Contrastingly, the decreased bioluminescence intensity, lung metastasis incidence, lung metastatic nodules number, and prolonged OS were observed in the MHCC97H-shETV1 group, compared with the MHCC97H-shContorl group (Fig. 2 C-H). In addition, in the subcutaneous xenograft model, we observed that stable overexpression of ETV1 increased the size, weight, and Ki-67 expression of tumors, whereas ETV1 knockdown decreased the growth of tumors and diminished the Ki-67( +) cells compared to the control group (Fig. S1 F-H). Taken together, our findings suggested that increased expression of ETV1 was correlated with poor prognosis in patients with HCC and enhanced HCC metastasis.

### Metastasis-related genes *PTK2* and *MET* are downstream targets of ETV1

**Fig. 3:**
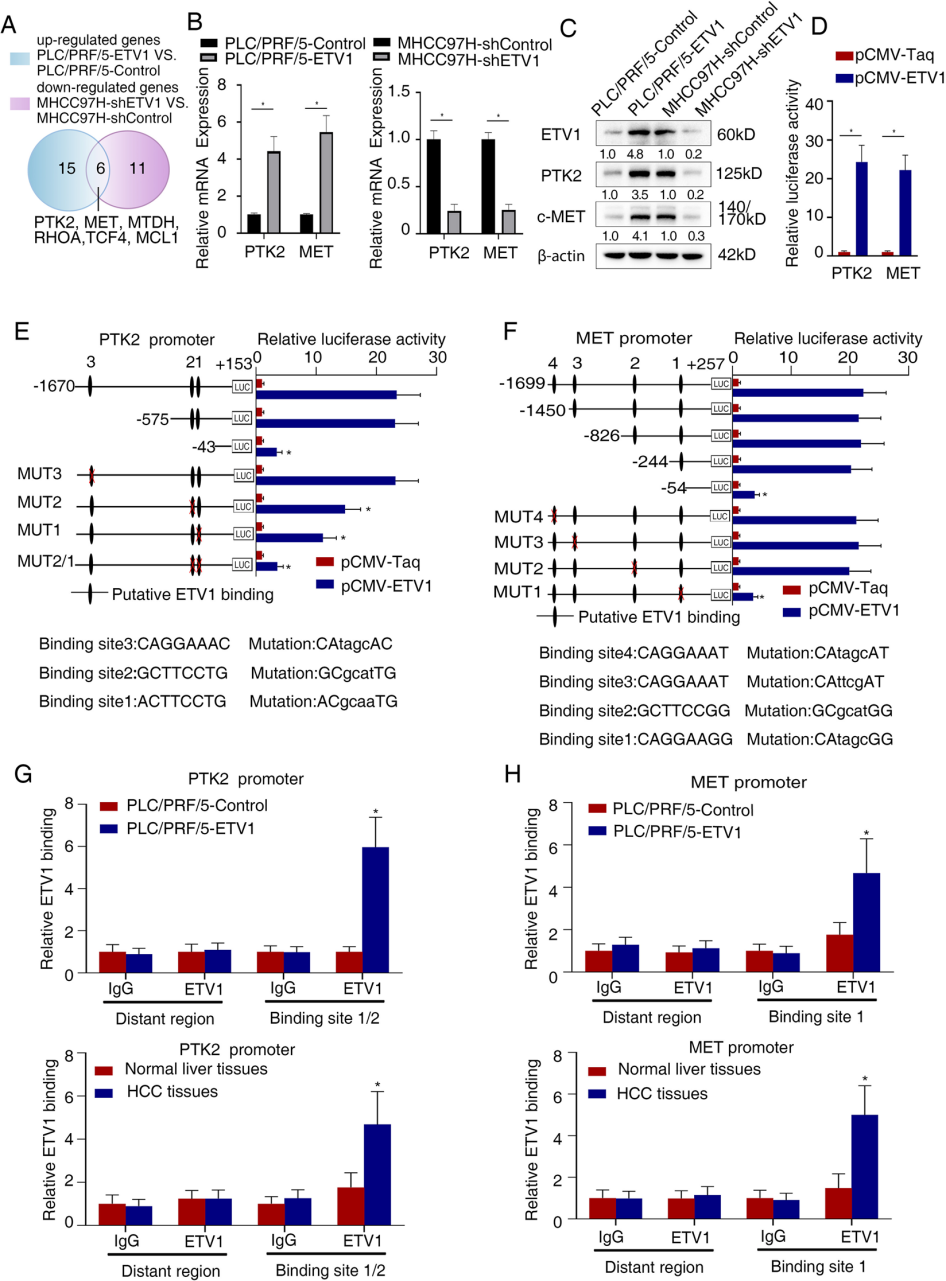
ETV1 transcriptionally upregulates PTK2 and c-MET expression. (**A**) Human Liver Cancer PCR Array was employed for detecting the upregulated genes in PLC/PRF/5-ETV1 and down-regulated genes in MHCC97H-shETV1 cells compared with controls (fold change ≥ 2.0). Overlapping genes were shown in the Venn diagram. (**B**-**C**) Compared with the controls, PTK2 and c-MET were upregulated when ETV1 was overexpressed in PLC/PRF/5 cells, and they were down-regulated when ETV1 was knocked down in MHCC97H cells, detected by qRT-PCR and western blotting. (**D**) Relative luciferase activity of PTK2 and MET promoter reporter vectors after cotransfection of pCMV-ETV1 and PTK2 or MET plasmid constructs in PLC/PRF/5 cells. (**E**-**F**) Relative luciferase activity of indicated PLC/PFR/5 cells cotransfected with pCMV-ETV1 and truncated or mutated luciferase constructs. (**G**-**H**) ChIP assay of ETV1 binding to the PTK2 or MET promoter in PLC/PRF/5 cells, as well as HCC specimens. * Represented p < 0.05. All data were displayed as Mean ± SD

Considering that ETV1-positive HCC displayed aggressive biological behavior, we next explored the mechanism underlying the role of ETV1 in facilitating HCC metastasis. Liver Cancer RT^2^ Profiler PCR Array was investigated with PLC/PRF/5-ETV1 cells, MHCC97H-shETV1 cells, and control cells. Among 84 liver cancer-related genes, there were 15 upregulated genes in PLC/PRF/5-ETV1 cells compared to control cells and 11 down-regulated genes in MHCC97H-shETV1 cells compared to control cells (Table S1 and S2). A total of 6 genes were overlapped, as shown in Fig. 3A. Among these, *PTK2* and *MET* were the most greatly changed genes and selected for further analysis. TCGA and GEO data indicated that there was a positive correlation between *ETV1* and *PTK2, ETV1* and *MET* (Fig. S1 I). Then we conducted RT-qPCR and western blotting to evaluate the impact of ETV1 on two candidates. Congruously, PTK2 and c-MET were significantly upregulated when ETV1 was overexpressed, and they were both down-regulated following ETV1 knockdown (Fig. 3B, C).

To further elucidate whether ETV1 regulates these two genes, luciferase reporter assays were performed. It indicated that exogenous expression of ETV1 enhanced the activity of *PTK2* and *MET* promoters (Fig. 3D). Several putative ETV1 binding sites were identified in *PTK2* promoter (*n* = 3) and *MET* promoter (*n* = 4) via bioinformatics analysis (Fig. S2 and S3). Then we generated a series of truncated reporters with deletions in the *PTK2* promoter sequence (Table S3). Luciferase reporter assays showed that deleting the sequence between -575 and -43 base pairs significantly decreased the activity of the *PTK2* promoter enhanced by ETV1 overexpression. Consistent with this, site-directed mutagenesis and luciferase assays indicated that mutation of the putative ETV1-binding site 1 and site 2 located between -575 to -43 base pairs decreased ETV1-meditated *PTK2* promoter activity (Fig. 3E). The same method was performed for the identification of the specific binding sites of ETV1 in the *MET* promoter. Through serial deletion and site-directed mutagenesis, putative binding site 1 located in the region between -244 and -54 bp was found to mediate ETV1-induced MET transactivation (Fig. 3F). Moreover, the direct binding of ETV1 to the promoter region of these two genes described above in HCC cells and specimens was validated by chromatin immunoprecipitation (ChIP) assay (Fig. 3G, H). Together, these data suggested that ETV1 was directly bound to the *PTK2* and *MET* promoters to transcriptionally upregulate their expression.

### ETV1 promotes HCC metastasis through upregulating PTK2 and c-MET expression

**Fig. 4:**
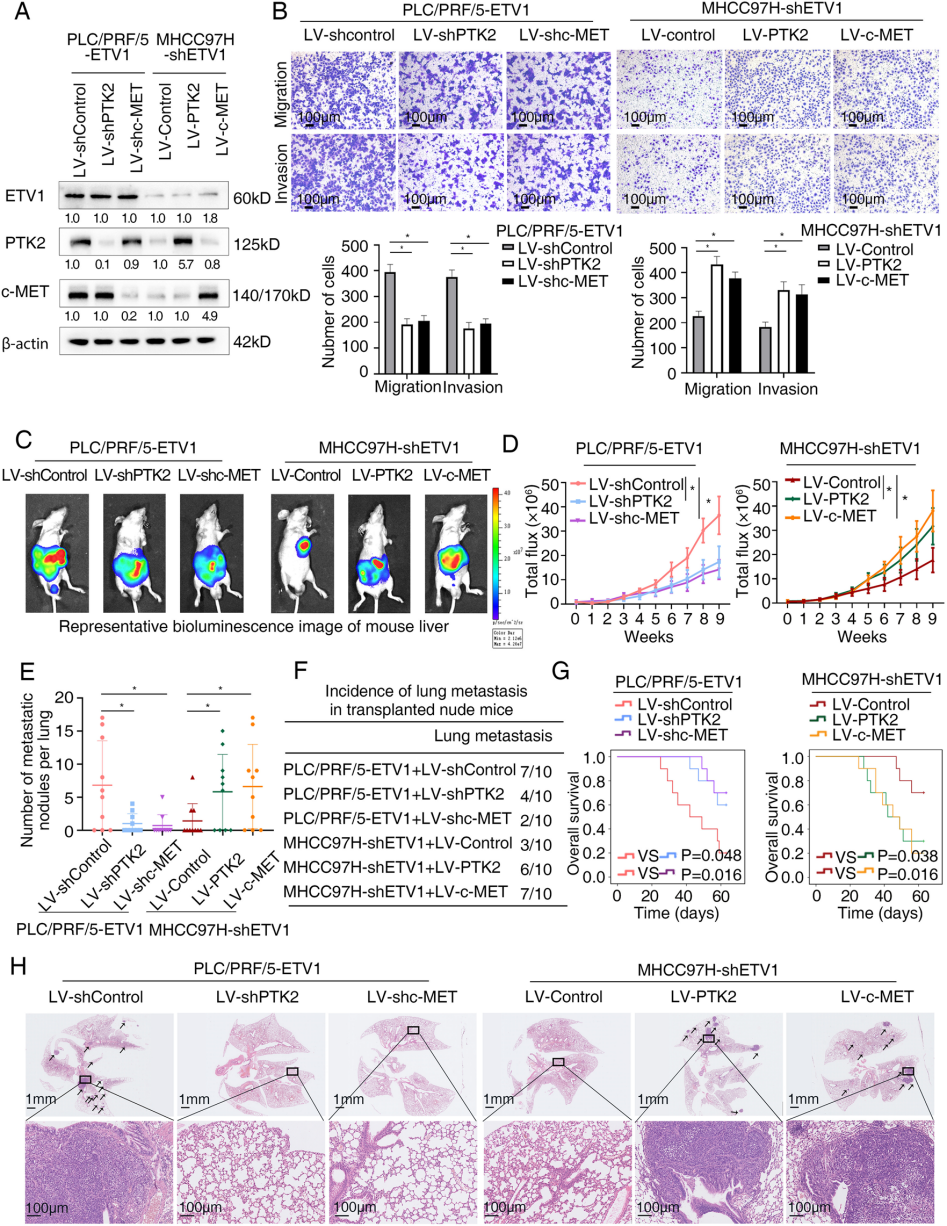
ETV1 promotes HCC metastasis by upregulating PTK2 and c-MET. (**A**) The levels of ETV1, PTK2, and c-MET in HCC cells after transfected with indicated lentivirus. (**B**) The migratory and invasive capacity were determined by transwell assays in the indicated HCC cells. (**C**-**H**) In vivo metastatic assay. (**C**) The representative BLI images in the liver were shown 9 weeks after implantation with indicated cells. (**D**) The bioluminescent signals were used to show the growth rate of liver tumors. (**E**) The number of metastatic lesions in the lung tissues. (**F**) The occurrence of lung metastasis. (**G**) The OS of different groups of nude mice. (**H**) Representative images of H&E staining of lung samples (indicated by arrowheads) from each group. * Represented p < 0.05. All data were displayed as Mean ± SD

PTK2, also known as FAK, is both a non-receptor tyrosine kinase and an adaptor protein with roles in cell migration, motility, invasion, and adhesion signaling [32]. It is overexpressed in multiple cancers and associated with cancer progression and metastasis [33]. In HCC, PTK2 overexpression is associated with capsular invasion, intrahepatic metastasis, and TNM stage in the detection of human samples [34]. c-MET, encoded by *MET*, plays a central role in promoting tumor invasive growth and driving cancer progression towards metastasis [35]. To address the effects of PTK2 and c-MET on ETV1-mediated HCC metastasis, we downregulated PTK2 and c-MET expression in PLC/PRF/5-ETV1 cell line and upregulated PTK2 and c-MET expression in MHCC97H-shETV1 cell line with lentiviral infection (Fig. S4A, B, Fig. 4A and Table S4). Compared to the controls, PTK2 or c-MET knockdown decreased the migratory and invasive ability of ETV1-overexpressing HCC cells, whereas PTK2 or c-MET overexpression increased the migratory and invasive ability reduced by the ETV1 down-regulation (Fig. 4B).

More importantly, the involvement of PTK2 and c-MET in ETV1-mediated HCC metastasis was further verified in vivo. As shown in Fig. 4 C-H, PTK2 or c-MET knockdown in PLC/PRF/5-ETV1 orthotropic group reduced bioluminescence intensity, lung metastasis incidence, and lung metastatic nodules number and extended the survival of mice compared with the control group. Reciprocally, the bioluminescence intensity, lung metastasis incidence, and lung metastatic nodules number were increased when PTK2 or c-MET overexpression compared to the control group. The survival of tumor-bearing mice was shortened via PTK2 and c-MET overexpression. Together, these findings indicated that PTK2 and c-MET were crucial to ETV1-mediated HCC metastasis.

Besides *PTK2* and *MET*, we detected the expression levels of four other overlapped genes. The mRNA and protein level of MTDH, RHOA, TCF4, MCL1 were up-regulated when ETV1 was overexpressed (Figure S5A, B). However, in transwell assays, knockdown of MTDH, RHOA, TCF4 and MCL1 slightly or moderately decreased migration and invasion ability increased by overexpression of ETV1 in PLC/PRF/5 cells (Figure S5C). These results indicated that MTDH, RHOA, TCF4 and MCL1 were not the main targets for ETV1-induced migration and invasion of HCC cells.

### HGF/c-MET axis upregulates ETV1 via ERK1/2 activation

Given that ETV1 was significantly upregulated in HCC and promoted the malignant progression of HCC, we sought to investigate the molecular mechanisms facilitating ETV1 upregulation in HCC. Since c-MET contributed to the aggressive peculiarities of ETV1-overexpressing HCC, its well-known ligand HGF attracted our attention. Binding to c-MET, HGF acts as a trigger for various cellular processes. In cooperation with GDNF, HGF induces ETV4 expression in spinal cord explants for recruitment of motor neurons, meanwhile, ETV4 is also required for the induction of c-Met expression by GDNF [36]. c-Met signaling regulates the expression the PEA3 factors to promote cell migration and invasion in gastric and lung cancer cells with MET-addicted [37]. HGF upregulates ETV5 to promote cell invasion of oral squamous cell carcinoma [38]. These studies suggested that the HGF/c-Met axis might be involved in regulation of PEA3 subfamily. We were interested in whether HGF is a regulator of ETV1 in HCC.

**Fig. 5:**
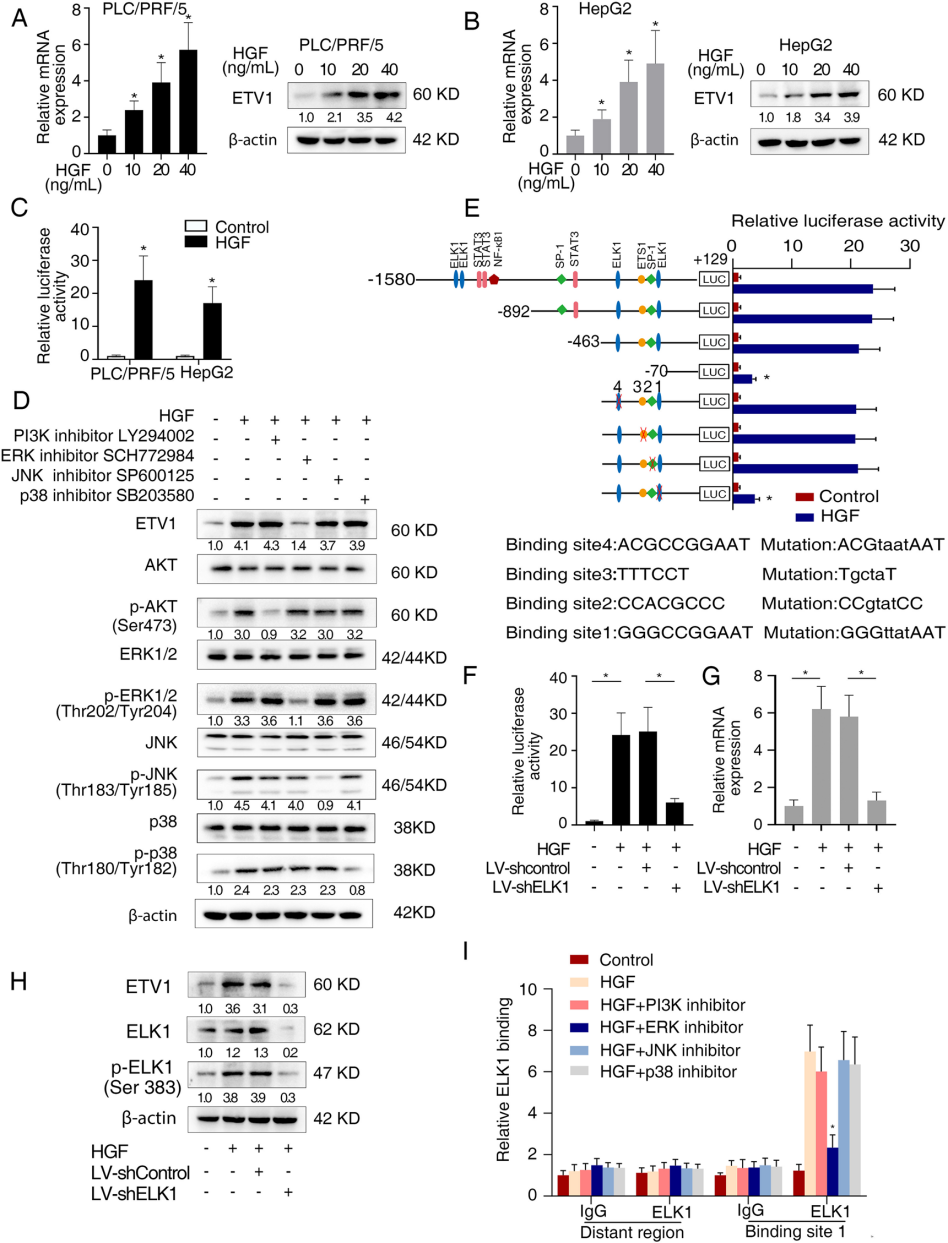
HGF/c-MET axis upregulates ETV1 via ERK1/2 activation. (**A**) The expression of ETV1 in PLC/PRF/5 cells treated with different concentrations of recombinant HGF. (**B**) The expression of ETV1 in HepG2 cells treated with different concentrations of HGF. (**C**) Relative luciferase activities of ETV1 promoter reporter vectors in indicated cells upon treatment with HGF. (**D**) Western blotting assays of ETV1, Akt, p-Akt, ERK1/2, p-ERK1/2, JNK, p-JNK, p38, p-p38, proceed with PI3K inhibitor (LY294002), ERK1/2 inhibitor (SCH772984), JNK inhibitor (SP600125), and p38 inhibitor (SB203580) upon HGF treatment in PLC/PRF/5 cells. (**E**) Relative luciferase reporter assays of PLC/PRF/5 cells transfected with serially truncated or mutant ETV1 promoter luciferase constructs treated with or without HGF. (**F**-**H**) The relative promoter luciferase activity, mRNA, and protein levels of ETV1 in the PLC/PRF/5 cells after transfection with shELK1 or control shRNA in the presence of HGF. (**I**) ChIP assays verified the direct binding of ELK1 to the ETV1 promoter in HCC cells. * Represented p < 0.05. All data were displayed as Mean ± SD

We treated PLC/PRF/5 cells with different concentrations of recombinant HGF. HGF increased the ETV1 mRNA and protein expression levels in a dose-dependent manner. Similar results were observed in HepG2 cells with relatively low ETV1 expression (Fig. 5A, B). Luciferase reporter assay demonstrated that *ETV1* promoter activity was augmented upon HGF treatment (Fig. 5C). HGF/c-MET axis activates several signaling cascades, including MAPK and PI3K/Akt. PI3K inhibitor LY294002, ERK1/2 inhibitor SCH772984, JNK inhibitor SP600125, and p38 inhibitor SB203580 were applied to treat PLC/PRF/5 cells. SCH772984 significantly diminished HGF-induced ETV1 expression, while other inhibitors did not have such effect (Fig. 5D). ERK1/2 was also knockdown with shRNA to determine its effect on ETV1 expression upon HGF treatment. As shown in Fig.S6A, knockdown of ERK1/2 decreased the ETV1 level upregulated upon HGF treatment. These data suggested that HGF upregulated ETV1 via ERK1/2 pathway.

To specify important *cis-*regulatory elements in the *ETV1* promoter, we analyzed the *ETV1* promoter and searched for putative transcription factor binding sites (Fig. 5E). We constructed a series of truncations/mutations of the *ETV1* promoter sequence (Fig. S7 and Fig. 5E). Promoter activity of the constructs induced by HGF was decreased between -463 bp to -70 bp, suggesting this region was vital to regulate the activity of *ETV1* promoter (Fig. 5E). Mutant constructs for the ELK1, ETS1, and SP-1 binding sites were generated using point mutagenesis. Of these mutant constructs, mutants of one ELK1 binding site, binding site 1, in this region showed a significant reduction in activity (Fig. 5E). Knockdown of ELK1 abolished the increased activity of *ETV1* promoter and the upregulated ETV1 expression level induced by HGF (Fig. 5F-H and Fig. S4C). Further, the ChIP assay validated that SCH772984 mitigated the binding of ELK1 to the *ETV1* promoter in PLC/PRF/5 cells treated by HGF, whereas other inhibitors had no such obvious effect (Fig. 5I). Moreover, we detected the serum HGF levels in the HCC patients by ELISA and detected the ETV1 levels in the tumor tissues of HCC patients by IHC (Fig. S8A). The correlation analysis showed that ETV1 score (0–12) positively correlated with the levels of serum HGF (*r* = 0.4970, *P* = 0.005) in the HCC patients (*n* = 30) (Fig. S8B). And positive correlations between *ETV1* and *HGF, ETV1* and *ELK1* expression were observed in TCGA and GEO databases (Fig. S8C, D). Accordingly, the ERK1/2-ELK1 cascade was responsible for an augmented level of ETV1 induced by HGF. HGF upregulated ETV1expression via c-MET-ERK1/2-ELK1 cascade, which in turn upregulated c-MET expression, forming a positive feedback.

### ETV1 is vital for HGF-mediated HCC invasion and metastasis

**Fig. 6:**
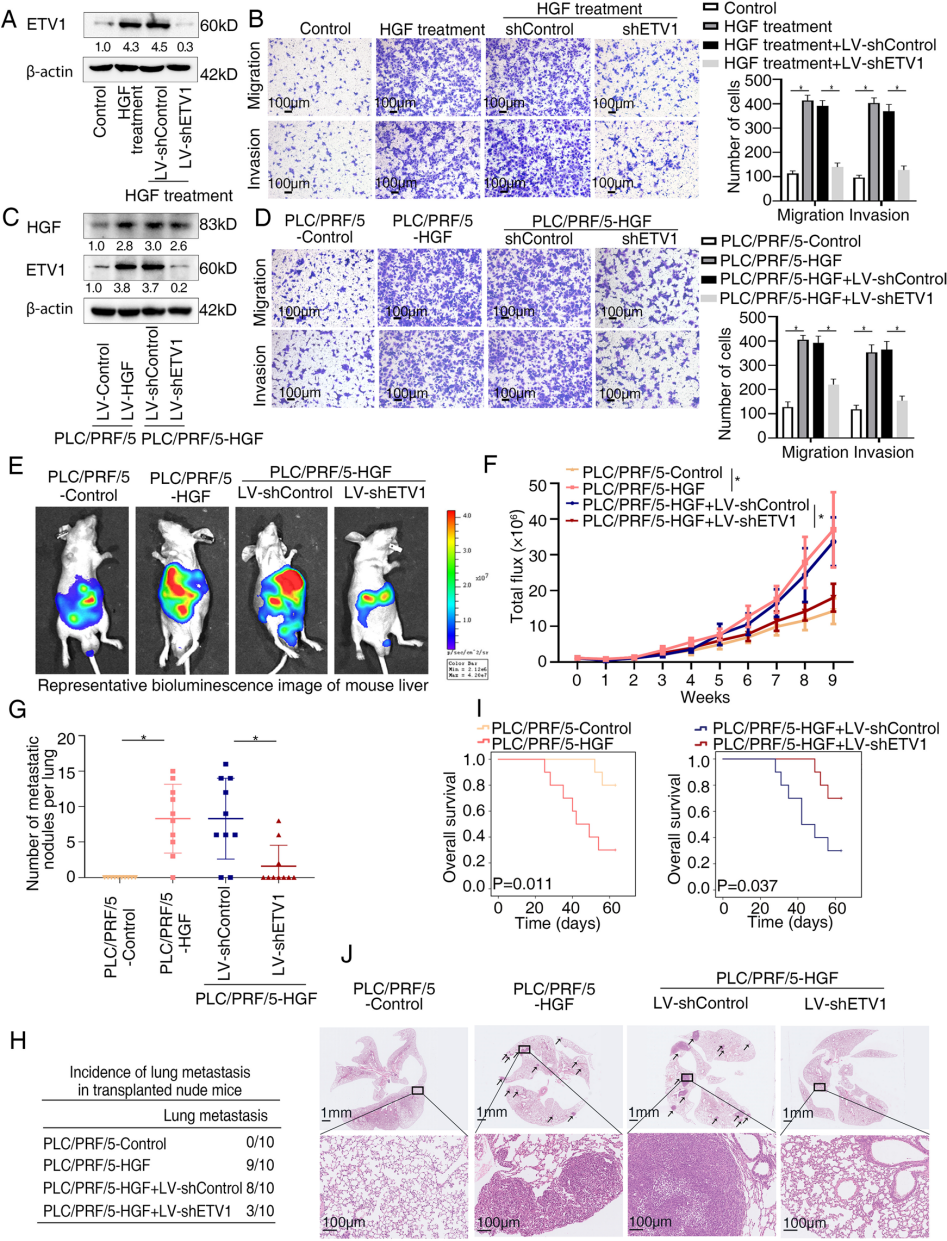
ETV1 is vital for HGF-mediated HCC invasion and metastasis. (**A**) The protein levels of ETV1 in the PLC/PRF/5 cells transfected with LV-sh Control or LV-shETV1 upon HGF treatment. (**B**) Transwell assays displayed the migratory and invasive capacity of the indicated cells upon HGF treatment. (**C**) The levels of HGF and ETV1 in the PLC/PRF/5-HGF cells transfected with LV-shControl or LV-shETV1. (**D**) Transwell assays displayed the migratory and invasive capacity of the indicated cells. (**E**-**J**) In vivo metastatic assay. (**E**) The representative BLI images in the liver were shown 9 weeks after implantation with indicated cells. (**F**) The bioluminescent signals were used to show the growth rate of liver tumors. (**G**) The number of metastatic lesions in the lung tissues. (**H**) The occurrence of lung metastasis. (**I**) The OS of different groups of nude mice. (**J**) Representative images of H&E staining of lung samples (indicated by arrowheads) from each group. * Represented p < 0.05. All data were displayed as Mean ± SD

In HCC, HGF is expressed by stromal cells or tumor cells and binds to its specific receptor, c-MET, to play a part in tumor onset, proliferation, invasion and metastasis [39]. The serum level of HGF is associated with extrahepatic metastasis [40, 41]. HGF induced by hepatectomy could promote metastasis of residual HCC cells [42]. Mechanistically, HGF regulates HCC invasion and metastasis by promoting EMT, activating signaling pathways, upregulating MMPs and so on [39]. Given the significance of HGF in HCC invasion and metastasis, we processed to test whether ETV1 contributes to HGF-induced HCC metastasis. Recombinant HGF was used to treat ETV1-knockdown PLC/PRF/5 cells (Fig. 6A). The promoting effects on cell migration and invasion enhanced by HGF were dampened when ETV1 was knockdown (Fig. 6B). Then we generated stable PLC/PRF/5-HGF cell line and knockdown ETV1 expression with shRNA. Exogenous HGF expression and knockdown of endogenous ETV1 in the indicated cells were confirmed by western blotting (Fig. 6C). Transwell assays demonstrated that downregulation of ETV1 inhibited the promoting effects of HGF-overexpressing on cell invasion and migration (Fig. 6D). Since ERK1/2 inhibitor inhibited ETV1 upregualtion induced by HGF (Fig. 5D), to better ascertain the role of ERK1/2-ETV1 in HGF-mediated HCC cell invasion and migration, we investigated the effect of ERK1/2 inhibitor SCH772984 on migration and invasion of PLC/PRF/5 cells mediated by recombinant HGF. The results exhibited that promoting effects on cell migration and invasion enhanced by HGF were dampened when treated with ERK1/2 inhibitor (Fig. S9A). Similarly, SCH772984 treatment inhibited the increase of cell migration and invasion induced by HGF overexpression (Fig. S9B).

Consistent with our data in vitro*,* the results of in vivo models exhibited that compared with the control group, the HGF-overexpressing group demonstrated dramatically increased bioluminescence intensity, lung metastasis incidence, and lung metastatic nodules number, as well as shortened overall survival. Contrastingly, these HGF-induced phenotypes were decreased when ETV1 was knocked down (Fig. 6E-J). Data above suggested that ETV1 was important for HGF-mediated HCC metastasis.

### ETV1 expression correlates with PTK2 and c-MET expression in HCC specimens

**Fig. 7:**
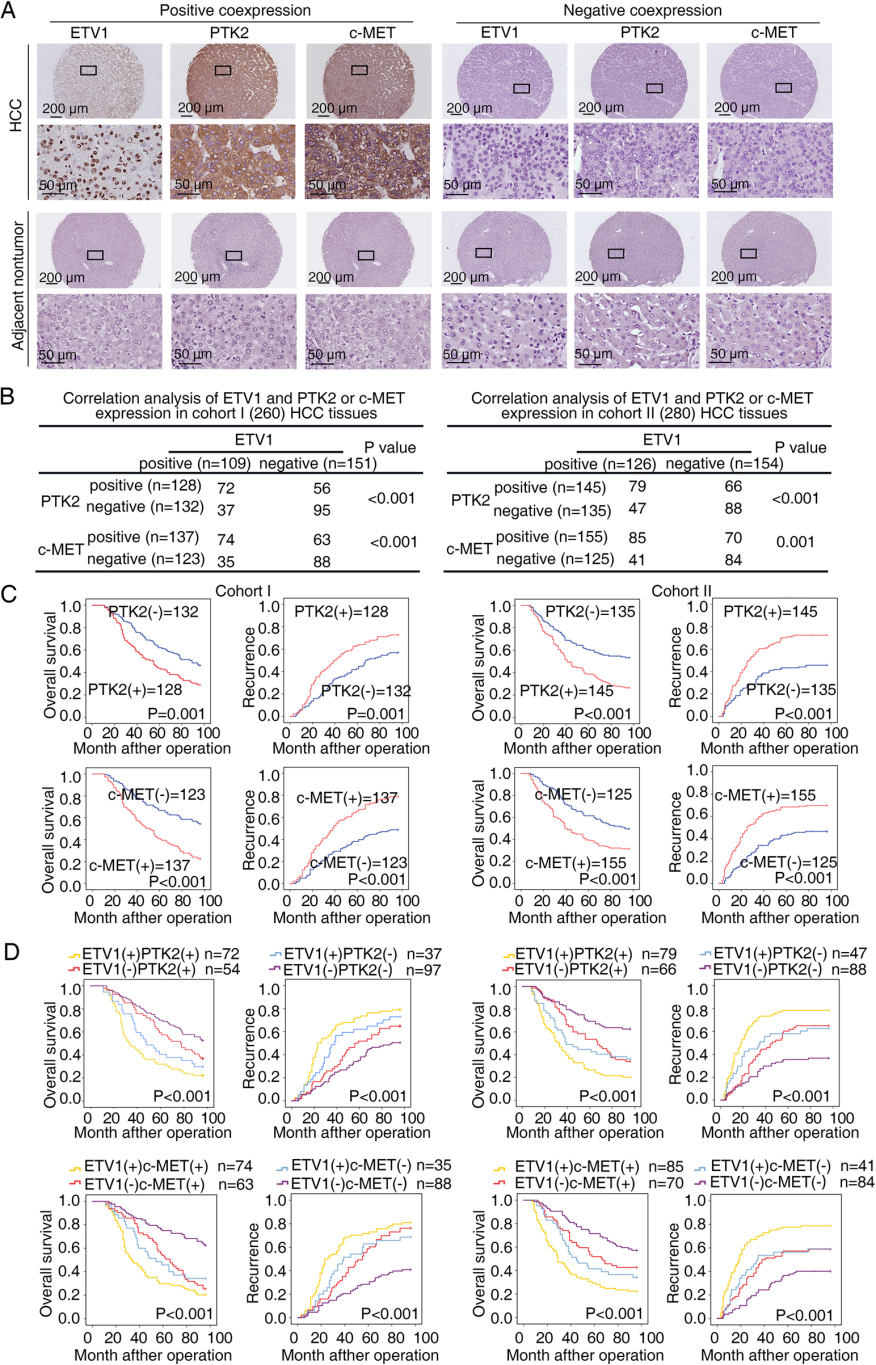
ETV1 expression correlates with PTK2 and c-MET expression in HCC specimens. (**A**) Representative IHC staining of ETV1, PTK2, and c-MET in primary HCC specimens and paired nontumorous specimens. (**B**) Association between ETV1 and PTK2 or c-MET expression in two HCC cohorts. (**C**-**D**) Kaplan-Meier curves showed the correlations between PTK2, c-MET, ETV1/PTK2, or ETV1/c-MET expression and OS or recurrence rates. * Represented p < 0.05. All data were displayed as Mean ± SD

On account of the contribution of ETV1 to HCC metastasis, we next performed IHC assays and correlation analysis to explore whether there is a clinical relevance of ETV1 with its two downstream targets. In two HCC patient cohorts (cohort I, *n* = 260; cohort II, *n* = 280), ETV1 expression was correlated with PTK2 and c-MET expression (Fig. 7A, B), and elevated expression of PTK2 or c-MET was associated with poor tumor differentiation, microvascular invasion, loss of encapsulation, and advanced TNM stage (Table S5 and S6). Moreover, patients with positive PTK2 or c-MET expression had higher recurrence rates and shorter OS than patients with negative PTK2 or c-MET expression (Fig. 7C). Subpopulations with ETV1/PTK2 or ETV1/c-MET coexpression had the highest recurrence rates and the shortest OS, as illustrated by Kaplan–Meier curves (Fig. 7D).

In addition, the mRNA and protein levels of ETV1, PTK2, and c-MET were detected in 20 cases of paired primary and metastatic specimens, and they were the highest in metastatic ones compared with primary HCC and adjacent nontumorous specimens (Fig. S10A-C).

### The combination of defactinib with capmatinib is effective in reducing ETV1-mediated HCC metastasis

**Fig. 8:**
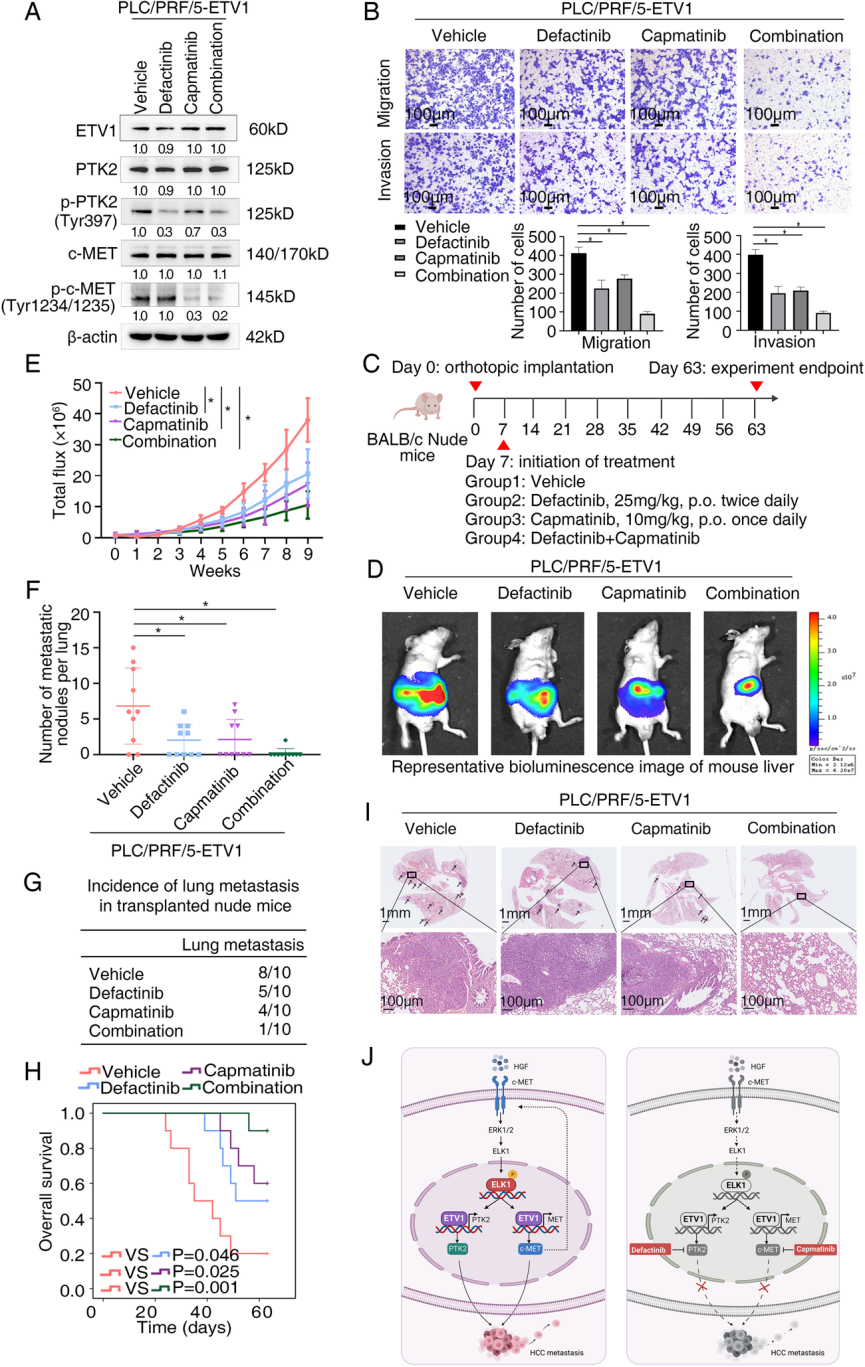
The combination of defactinib with capmatinib is effective in reducing ETV1-mediated HCC metastasis (**A**) The levels of ETV1, p-PTK2 (Tyr397), PTK2, p-c-MET (Tyr1234/1235), c-MET in PLC/PRF/5-ETV1 cells processed with a single agent or the combination of defactinib and capmatinib. (**B**) HCC cell invasion and migration was evaluated by transwell assay. The combination treatment of defactinib and capmatinib showed the greatest inhibitory effect among all the groups. (**C**) Schematic diagram of treatment in nude mice of four groups. (Group1: Vehicle, Group2: Defactinib, Group3: Capmatinib, Group4: Defactinib combined with Capmatinib) (**D**-**I**) In vivo metastatic assay. (**D**) The representative BLI images in the liver were shown 9 weeks after implantation with indicated cells. (**E**) The bioluminescent signals were used to show the growth rate of liver tumors. (**F**)The number of metastatic lesions in the lung tissues. (**G**) The occurrence of lung metastasis. (H) The OS of different groups of nude mice. (**I**) Representative images of H&E staining of lung samples (indicated by arrowheads) from each group. * Represented p < 0.05. All data were displayed as Mean ± SD. (**J**) Diagram illustrating the mechanisms underlying how ETV1 promotes HCC metastasis and a potential combination strategy. ETV1 mediates HCC metastasis through increasing PTK2 and c-MET expression. HGF upregulates ETV1 expression via c-MET-ERK1/2-ELK1, creating a positive feedback loop that continuously stimulates HCC development. PTK2 inhibition in combination with c-MET inhibition markedly mitigates ETV1-mediated HCC metastasis

Defactinib is a leading PTK2 inhibitor investigated in several clinical trials in combination with other agents. It functions via inhibition of phosphorylation of Tyr-397, a critical target of PTK2 activation [32, 43]. Capmatinib is a highly selective tyrosine kinase inhibitor targeting c-MET. It was approved by the FDA for patients with metastatic non-small cell lung cancer harboring *MET* exon 14 skippings based on the GEOMETRY mono-1 study in 2020 [44]. Given the significance of PTK2 and c-MET in ETV1-mediated HCC metastasis, we next evaluated the antitumor efficacy of the two inhibitors mentioned above. The efficacy of defactinib plus capmatinib was verified by decreased expression of p-PTK2 (Tyr397) and p–c-MET (Tyr1234/1235) (Fig. 8A). Intriguingly, transwell assays exhibited that the combination treatment of defactinib and capmatinib significantly decreased migratory and invasive ability of PLC/PRF5-ETV1 cells, whereas treatment with defactinib or capmatinib alone played a partial inhibitory effect (Fig. 8B). Similar results were observed in the in vivo models. Compared to the control group, treatment with defactinib or capmatinib alone partially reduced lung metastasis incidence together with lung metastatic nodules number and improved the OS to some extent, whereas the combined treatment significantly decreased bioluminescence intensity, lung metastasis incidence, lung metastatic nodules number, and prolonged the survival of the nude mice (Fig. 8C-I). Altogether, these findings demonstrated that the combination therapy with PTK2 inhibitor defactinib and c-MET inhibitor capmatinib inhibited HCC metastasis induced by ETV1 expression.

## Discussion

Metastasis is the main factor of poor prognosis in patients with HCC [2]. It is of great significance to gain insight into its mechanisms. As a member of ETS/PEA3 family, ETV1 has been well documented to represent oncogenic drivers across several tumors including prostate cancer, Ewing sarcoma and GIST [21, 45, 46]. Besides, authors have reported its clinical significance. High ETV1 levels are correlated with shorter prostate-specific antigen (PSA) recurrence in prostate cancer, while nuclear overexpression of ETV1 is associated with longer overall survival in esophageal adenocarcinoma [47, 48]. Functionally, ETV1 influences tumor progression by inducing EMT, promoting cell migration and invasion, regulating cell cycle and apoptosis, as well as mediating chemotherapy resistance [12]. However, its expression and role in HCC remain elusive. In this study, we found the elevated expression of ETV1 in HCC specimens and a close correlation between high ETV1 expression and advanced TNM stage, microvascular invasion, and loss of encapsulation. More crucially, multivariable Cox regression implied that elevated expression of ETV1 was a valuable indicator for poor prognosis in HCC. Compared with normal specimens, metastatic specimens had higher ETV1 levels than primary specimens. By transwell assays and the orthotopically metastatic model, we proved that ETV1 was critical for invasion and metastasis of HCC. Altogether, both clinical findings and functional studies suggested that ETV1, a transcription factor, whose expression was associated with prognosis of HCC patients, displayed a potent promoting effect on HCC metastasis.

ETV1 binds to many target genes and plays a role in tumor progression and metastasis by regulating expression of them. It has previously been shown to confer the aggressive biologic behavior of tumors via inhibition of checkpoint kinase 1 (*CHK1*), upregulation of EMT-associated genes such as *TWIST1*, *SNAIL*, *HAS2*, pro-metastatic genes such as *MMP1/7*, *SPARC*, methyltransferase *METTL3* [20, 49–53]. Herein, we uncovered that PTK2 and c-MET were transcriptional targets of ETV1. PTK2 is an important signaling “hub”, primarily regulating adhesion signaling and cell migration, as well as cell survival under stress [32]. In HCC, PTK2 expression is frequently increased and is responsible for HCC cell invasion and migration, partially through regulating MMP2 and MMP9 [54, 55]. In particular, studies reported that elevated PTK2 and p-PTK2 Tyr397 levels are correlated with vascular invasion and intrahepatic metastasis, offering a promising target to treat HCC [54, 56]. c-MET is known to exert oncogenic functions in HCC [22]. It is reported that MET-regulated gene expression signature has a close correlation with increased vascular invasion and microvessel density in HCC patients [57]. c-MET regulates cell migration and invasion through activating its downstream effector including RAS/MAPK, PI3K/Akt and RAC1/CDC42 pathways. It can also promote HCC metastasis through regulating mitochondrial fission [58]. Accordingly, both PTK2 and c-MET play significant roles in HCC invasion and metastasis. Herein, PTK2 and c-MET were identified as transcriptional targets of ETV1. PTK2 or c-MET knockdown impeded ETV1-mediated HCC metastasis. Reciprocally, PTK2 or c-MET overexpression rescued HCC metastasis inhibited by ETV1 knockdown. In two HCC patient cohorts, ETV1 expression was positively associated with PTK2 and c-MET expression. It was noted that coexpression of ETV1/PTK2 or ETV1/c-MET subpopulations displayed the poorest OS and the highest recurrence rate. These results verified that PTK2 and c-MET were required for the promoting effect of ETV1 on HCC metastasis.

The regulatory mechanism of ETV1 overexpression in human HCC remains unknown. Prior studies have reported that ETV1 expression can be regulated by several ways. FOXF1 transcriptionally regulates ETV1 mainly through enhancer binding. FOXF1 co-localizes with ETV1 at enhancers to regulate the ETV1-dependent GIST-lineage specific transcriptome [59]. Constitutive photomorphogenetic 1 (COPI) and capicua (CIC) have been identified as repressors of ETV1. COPI regulates the expression of ETV1 at post-translational level by directly binding to its N-terminus and leading to the ubiquitination degradation [60]. CIC is a transcriptional repressor of the HMG-box family which binds specific DNA sites in target genes [61]. The well-characterized mammalian targets of CIC are ETV1/4/5. Dysregulation of CIC induced de-repression of ETV1/4/5 was found to negatively affect patient prognosis or drive aggressive phenotypes [62]. Moreover, ETV1 is reported to be significantly upregulated by FGF2 treatment and downregulated by TGF-β1, thus generating distinct cancer-associated fibroblasts populations to promote skin squamous cell carcinoma development [63]. In HCC, we observed that HGF upregulated ETV1 in a dose-dependent way. Previous study reported that the ERK1/2 and p38 inhibitors affect the ETV1 level in colorectal cancer cells [64]. Our results showed that inhibition of the ERK1/2 pathway diminished HGF-induced ETV1 expression, whereas other inhibitors had no such effect. The overexpression of ETV1 upregulated the expression of c-MET, which in turn enhanced HCC sensitivity to HGF stimulation, thus creating a positive feedback loop. HGF-c-MET axis plays a part in augmenting HCC angiogenesis, invasion, and metastasis [41]. Consistent with this, our data suggested that migration and invasion of HCC cells were enhanced under HGF treatment, whereas ETV1 knockdown decreased the migration and invasion capability of HCC cells enhanced by HGF.

To find an effective way of inhibiting HCC metastasis mediated by ETV1, we focused on pharmacological inhibitors of PTK2 and c-MET. Due to the significance of PTK2, PTK2 inhibitors are being actively developed, showing well tolerability [65]. Defactinib (VS-6063), an ATP-competitive PTK2 inhibitor, functions via inhibiting phosphorylation of Tyr397 and downstream pathways [66]. Defactinib can impede the initiation of liver cancer and ameliorate the efficacy of sorafenib in HCC [67]. Nonetheless, one phase II study showed that defactinib has only modest antitumor activity [68]. In our study, treatment with defactinib alone partially reduced migration and invasion capability of PLC/PRF/5-ETV1 HCC cells and slowed down HCC growth. We observed the similar effect of c-MET inhibitor on HCC metastasis. Given the role of PTK2 and c-MET in ETV1-meditated metastasis and the discovery of the HGF-ETV1-c-MET loop, we speculated that c-MET inhibitors might cooperate with PTK2 inhibitors in targeting ETV1-overexpressing HCC. c-Met inhibitors have shown antitumor potential in preclinical models, and many of them are currently in clinical trials for cancer [69]. Regrettably, the benefits of these drugs applied alone have not been as impressive as initially hoped [5]. Capmatinib is a highly potent MET inhibitor with manageable safety, found to be highly selective for c-MET over other kinases. Tumor regression was observed on a liver cancer xenograft model treated with capmatinib [70]. Our data illustrated that the combination of defactinib plus capmatinib conspicuously promoted anti-HCC efficacy in vitro and in vivo, and the combined treatment may have utility for HCC patients with ETV1 overexpression. However, this aspect warrants further investigation.

In conclusion, we demonstrated that ETV1 was frequently increased in HCC, mainly promoted by HGF. HGF/c-MET axis enhanced ETV1 expression via ERK1/2-ELK1 pathway, leading to PTK2 and c-MET upregulation and subsequent augmented HCC metastasis. Targeting the HGF-ETV1-PTK2/c-MET axis may provide evidence for developing a novel treatment strategy to ameliorate HCC progression.

## Conclusions

Our study shows that increased ETV1 expression functionally contributes to HCC metastasis. In addition, ETV1 abundance correlates with patient survival and recurrence and thus may have prognostic value for patients with HCC. Mechanistically, ETV1 upregulates PTK2 and c-MET expression, and HGF upregulates ETV1 expression through c-MET-ERK1/2-ELK1 pathway, forming a positive feedback loop. Combined treatment of defactinib and capmatinib significantly suppressed HCC metastasis, which may provide a potential targeting strategy for HCC treatment.

## Supplementary Information

**Additional file 1:** **Supplementary materials. FigureS1. **(A) *ETV1*expression in LIHC and correlation of *ETV1* expression with overallsurvival were analyzed in LIHC according to the data of The Cancer Genome Atlas(TCGA). (B) CellCounting Kit-8 (CCK8) assay assessing the cell proliferation of theETV1-overexpressing PLC/PRF/5 cells and ETV1-knockdown MHCC97H cells. (C) Colony formation assay showing the proliferationof the indicated HCC cells. Therepresentative photos were shown and the cell numbers were quantified. (D-F) Tumorgrowth of the indicated HCC cells was assessed by subcutaneous xenograft tumormodels. The tumor volume and weight were shown in (D) and (E), the representative images of Ki67 were shownin (F). *n* = 5 in each group. (G) The correlation between ETV1 expression and PTK2 or MET expression inTCGA-LIHC and GEO database. *p < 0.05, ****p < 0.0001. Data were shown as Mean ± SD. **Figure S2. **ETV1 binding sites withinthe promoter regions of *PTK2*. Thesequences highlighted in yellow represent the three binding sites of ETV1 onthe PTK2 promoter, and the arrow represents the transcription initiation sites.The   mutagenesis of the promoter sequencewere annotated. **Figure S3. **ETV1binding sites within the promoter regions of *MET*. The sequenceshighlighted in yellow represent the four binding sites of ETV1 onthe *MET *promoter, and the arrow represents the transcription initiation sites.The mutagenesis of the promoter sequence were annotated. **Figure S4. **(A**-**C) Western blotverifying PTK2 and MET knockdown effect in PLC/PRF/5-ETV1 cells and ELK1knockdown effect in PLC/PRF/5 cells. **FigureS5. **(A) The expression levels of MTDH, RHOA, TCF4 and MCL1 were determined in the indicated cells by real-time PCR. (B) Westernblotting assays of MTDH, RHOA, TCF4 and MCL1 in the indicated cells transfected with lentivirus. (C) The migratingand invasive capability of the indicated cells was determined via transwellassay. **Figure S6.** (A) The level of ETV1 in the PLC/PRF/5 cells upon HGFtreatment with/without ERK1/2 knockdown. **Figure S7. **Transcription factors binding siteswithin the promoter regions of *ETV1*. The sequences highlighted in blue represent the four binding sites ofELK1 on the *ETV1* promoter. The yellow highlighted sequences representthe one binding site of ETS1 onthe *ETV1* promoter. The sequenceshighlighted in grey represent the binding site of SP1 on the *ETV1*promoter. The red highlighted sequences represent the binding site of NF-ΚB1 onthe *ETV1* promoter. The pink highlighted sequences represent the bindingsequence of STAT3 on the *ETV1* promoter. The arrows representtranscription start sites. The mutagenesis of the promoter sequence wereannotated. **Figure S8. **(A)Representative IHC staining of ETV1 is shown. (B) Pearsoncorrelation analyses between ETV1 IHC score and the levels of serum HGF in HCCpatients. *n*=30. (C) The correlation between ETV1 expression and HGF expression inTCGA-LIHC and GEO database.(D) The correlation between ETV1expression and ELK1 expression in TCGA-LIHC and GEO database. **Figure S9. **Effectof ERK1/2 inhibitor on HGF-mediated HCC cell migration and invasion. (A)Transwell assays displayed the migratory and invasive capacity of theindicated cells upon HGF treatment. (B) Transwell assays displayed themigratory and invasive capacity of the indicated cells. **Supplementary Table S1.** List of genesdifferentially expressed in PLC/PRF/5-ETV1 versus PLC/PRF/5-Control cells usinga human liver cancer PCR array. **Supplementary Table S2.** List of genes differentially expressed in MHCC97H-shETV1 versusMHCC97H-shControl cells using a human liver cancer PCR array. **Supplementary Table S3.** Primer sequences usedin the study. **Supplementary Table S4. **Knockdown shRNA sequencesused in this study. **Supplementary Table S5.** Correlation between PTK2 expression and clinicopathologicalcharacteristics of HCCs in two independent cohorts of human HCC tissues. **Supplementary Table S6.** Correlationbetween c-MET expression and clinicopathological characteristics of HCCs in twoindependent cohorts of human HCC tissues.

## Data Availability

The data supporting our conclusion were obtained from the TCGA database and GEO database.

## Abbreviations

ETS: E-twenty-six transformation-specific
BLI: Bioluminescence imaging
ChIP: Chromatin immunoprecipitation
c-MET: Tyrosine protein kinase Met
HCC: Hepatocellular carcinoma
H&E: Hematoxylin and eosin
HGF: Hepatocyte growth factor
IHC: Immunohistochemical
MAPK: Mitogen-activated protein kinase
OS: Overall survival
PTK2: Protein tyrosine kinase 2
RT-qPCR: Quantitative reverse transcription-polymerase chain reaction
shRNA: Short hairpin RNA
TNM: Tumor-nodule-metastasis
