# Supplementary material for: HGF-mediated elevation of ETV1 facilitates hepatocellular carcinoma metastasis through upregulating PTK2 and c-MET
Source: J Exp Clin Cancer Res. 2022 Sep 16;41:275. doi: 10.1186/s13046-022-02475-2 (PMC9479266; doi:10.1186/s13046-022-02475-2)
Supplement: Supplementary file 1 — Additional file 1: Supplementary materials. FigureS1. (A) ETV1expression in LIHC and correlation of ETV1 expression with overallsurvival were analyzed in LIHC according to the data of The Cancer Genome Atlas(TCGA). (B) CellCounting Kit-8 (CCK8) assay assessing the cell proliferation of theETV1-overexpressing PLC/PRF/5 cells and ETV1-knockdown MHCC97H cells. (C) Colony formation assay showing the proliferationof the indicated HCC cells. Therepresentative photos were shown and the cell numbers were quantified. (D-F) Tumorgrowth of the indicated HCC cells was assessed by subcutaneous xenograft tumormodels. The tumor volume and weight were shown in (D) and (E), the representative images of Ki67 were shownin (F). n = 5 in each group. (G) The correlation between ETV1 expression and PTK2 or MET expression inTCGA-LIHC and GEO database. *p < 0.05, ****p < 0.0001. Data were shown as Mean ± SD. Figure S2. ETV1 binding sites withinthe promoter regions of PTK2. Thesequences highlighted in yellow represent the three binding sites of ETV1 onthe PTK2 promoter, and the arrow represents the transcription initiation sites.The mutagenesis of the promoter sequencewere annotated. Figure S3. ETV1binding sites within the promoter regions of MET. The sequenceshighlighted in yellow represent the four binding sites of ETV1 onthe MET promoter, and the arrow represents the transcription initiation sites.The mutagenesis of the promoter sequence were annotated. Figure S4. (A-C) Western blotverifying PTK2 and MET knockdown effect in PLC/PRF/5-ETV1 cells and ELK1knockdown effect in PLC/PRF/5 cells. FigureS5. (A) The expression levels of MTDH, RHOA, TCF4 and MCL1 were determined in the indicated cells by real-time PCR. (B) Westernblotting assays of MTDH, RHOA, TCF4 and MCL1 in the indicated cells transfected with lentivirus. (C) The migratingand invasive capability of the indicated cells was determined via transwellassay. Figure S6. (A) The level of ETV1 in the PLC/PRF/5 cells upon HGFtreatment with [file 13046_2022_2475_MOESM1_ESM.docx]

**Supplementary materials**

**Methods**

**Cell lines and culture**

PLC/PRF/5, HepG2, SNU423, Hep3B, SNU398, SNU449, and SNU387 were purchased from the American Type Culture Collection (ATCC, MD, USA). Huh7 was purchased from the Stem Cell Bank, Chinese Academy of Sciences. MHCC97H, HCCLM3, and HCCLM6 were kindly provided by Dr. Tang ZY (Liver Cancer Institute, Zhongshan Hospital, Fudan University, Shanghai, China). All the cell lines were authenticated by short tandem repeats (STRs) DNA profiling. Additionally, all the cell lines were checked by the MycoAlert Mycoplasma detection kit. Cells were cultured in Dulbecco’s Modified Eagle Medium (DMEM, GIBCO, CA, USA) containing 10% fetal bovine serum (FBS) (GIBCO, CA, USA) in 5% CO2 at 37 °C.

**Patients and follow-up**

This study was approved by the Ethics Committee of Tongji Medical College. Cohort I included 260 adult patients with HCC who underwent curative resection between 2003 and 2005 at the Tongji Hospital of Tongji Medical College (Wuhan, China). Cohort II included 280 adult patients with HCC who underwent curative resection between 2006 and 2008 at the Tongji Hospital of Tongji Medical College (Wuhan, China). A preoperative clinical diagnosis of HCC was based on the diagnostic criteria of the American Association for the Study of Liver Diseases. The inclusion criteria were as follows: (a) distinctive pathologic diagnosis; (b) no preoperative anticancer treatment or distant metastases; (c) curative liver resection; and (d) complete clinicopathologic and follow-up data. The differentiation statuses were graded according to the method of Edmondson and Steine. The pTNM classification for HCC was based on The American Joint Committee on Cancer/International Union Against Cancer staging system (6th edition, 2002). Follow-up data were summarized at the end of December 2013 (Cohort I) and December 2016 (Cohort II, range 4-96 months), respectively. The patients were evaluated every 2-3 months during the first 2 years and every 3-6 months thereafter. All follow-up examinations were performed by physicians who were blinded to the study. During each check-up, the patients were monitored for tumor recurrence by measuring the serum AFP levels and by performing abdominal ultrasound examinations. Computed tomography and/or magnetic resonance imaging examination was performed every 3-6 months, together with a chest radiographic examination. The diagnostic criteria for HCC recurrence were the same as the preoperative criteria. The time to recurrence and overall survival were the primary endpoints. The time to recurrence was calculated from the date of resection to the date of a diagnosis with tumor recurrence. The overall survival was calculated from the date of resection to the date of death or of the last follow-up.

In addition, 10 normal liver tissues, 50 pairs of fresh HCC tissues and adjacent nontumor tissue samples, and 20 pairs of fresh metastatic and matched primary HCC tissue and adjacent nontumor tissue samples were collected after surgical resection and were used for further investigations.

**Construction of tissue microarrays and immunohistochemistry score**

HCC samples and the corresponding adjacent liver tissues were used to construct a tissue microarray (Shanghai Biochip Co., Ltd. Shanghai, China). IHC analyses were performed by two independent observers who were blinded to the clinical outcome. The immunostaining intensity was scored on a scale of 0 to 3: 0 (negative), 1 (weak), 2 (medium), or 3 (strong). The percentage of positive cells was evaluated on a scale of 0 to 4: 0 (negative), 1 (1%-25%), 2 (26%-50%), 3 (51%-75%), or 4 (76%-100%). The final immuno-activity scores were calculated by multiplying the above two scores, resulting in an overall score ranges from 0~12. Each case was ultimately considered “negative” if the final score ranges from 0~3 and “positive” if the final score ranges from 4~12 as described previously.

**Western Blotting Analysis**

Proteins from lysed cells were fractionated by SDS-PAGE and transferred to nitrocellulose membranes. Nonspecific binding sites were blocked with 5% BSA in TBST (120 mM Tris–HCl (pH 7.4), 150 mM NaCl, and 0.05% Tween 20) for 2 h at room temperature. Blots were incubated with a specific antibody overnight at 4 °C. Western blotting of β-actin on the same membrane was used as a loading control. The membranes were then washed with TBST 3 times and incubated with an HRP-conjugated secondary antibody. Proteins were visualized using an ImmobilonTM Western Chemiluminescent HRP substrate (Millipore, MA, USA).

The primary antibodies used in western blotting were listed below.

| Antibodies | Source | Concentration |
| --- | --- | --- |
| anti-ETV1(ER81) | Ruiying, RLT1604 | 1:500 |
| anti-PTK2 | Cell Signaling Technology, #3285 | 1:1000 |
| anti-c-MET | Cell Signaling Technology, #8198 | 1:1000 |
| anti-ELK1 | Proteintech, 27420-1-AP | 1:2000 |
| anti-p-ELK1 | Cell Signaling Technology, #9181 | 1:1000 |
| anti-AKT | Cell Signaling Technology, #4685 | 1:1000 |
| anti-pAKT | Cell Signaling Technology, #4060 | 1:2000 |
| anti-ERK,  anti-P38,  anti-JNK | Cell Signaling Technology, #9926 | 1:2000  1:1000  1:1000 |
| anti-pERK,  anti-pP38,  anti-pJNK | Cell Signaling Technology, #9910 | 1:2000  1:1000  1:1000 |
| anti-HGF | Abcam, ab83760 | 1:1000 |
| anti-p-c-MET | Cell Signaling Technology, #3077 | 1:1000 |
| anti-pPTK2 | Cell Signaling Technology, #3283 | 1:1000 |
| anti-β-actin | Proteintech, 66009-1-Ig | 1:2000 |

**Quantitative reverse-transcription PCR (RT-qPCR)**

According to the manufacturer’s protocol, total RNA was extracted with TRIzol Reagent (TaKaRa, Japan) and reverse-transcribed with the PrimeScript RT Reagent Kit (TaKaRa, Japan). Quantitative RT-PCR was performed on an ABI QuantStudio 3 (Applied Biosystems, MA, USA) with SYBR Premix ExTaq (TaKaRa, Japan). The cycling parameters were as follows: 95 °C for 5 s and 60 °C for 30 s for 40 cycles. The melting curve and the Ct value were analyzed. The 2^–ΔΔCt^ method was used to determine relative fold changes in target gene expression in cell lines, which was normalized to expression levels in corresponding control cells (defined as 1.0). The equation used was 2^–ΔΔCt^ (ΔCt = Ct^target^ – Ct^ACTB^; ΔΔCt = ΔCt^expressing vector^ – ΔCt^control vector^). When calculating relative expression levels in surgically extracted HCC samples, relative fold changes in target gene expression were normalized to expression values in normal liver tissues (defined as 1.0) using the following equation: 2 ^–ΔΔCt^ (ΔΔCt = ΔCt^tumor^ – ΔCt^nontumor^). All experiments were performed in [triplicate](file:///D:/Apps/youdaocidian/Dict/8.9.6.0/resultui/html/index.html#/javascript:;). The primer sequences were listed in Supplementary Table S3.

***In Vitro* Migration and Invasion Assays**

The migratory and invasive ability of HCC cells were [evaluate](file:///D:/Apps/youdaocidian/Dict/8.9.6.0/resultui/html/index.html#/javascript:;)d using transwell inserts with an 8-µm pore size (Corning, NY, USA). DMEM supplemented with 10% FBS was added to the bottom chamber. Matrigel (50 µl, diluted 1:8 with DMEM, Corning, [New](file:///D:/Apps/youdaocidian/Dict/8.9.6.0/resultui/html/index.html#/javascript:;) [York](file:///D:/Apps/youdaocidian/Dict/8.9.6.0/resultui/html/index.html#/javascript:;), USA) was coated on the top chambers and dried for invasion assay. 5 × 10^4^ (migration assay) and 1 × 10^5^ (invasion assay) cells were seeded in the top chamber in serum-free medium and were cultured in 5% CO2 at 37 °C for 24 h and 72 h, [respectively](file:///D:/Apps/youdaocidian/Dict/8.9.6.0/resultui/html/index.html#/javascript:;). Cells that migrated or [invade](file:///D:/Apps/youdaocidian/Dict/8.9.6.0/resultui/html/index.html#/javascript:;)d the lower surface of the membrane were fixed, stained, and imaged. The cell numbers from five fields per membrane of three inserts were used for statistical analysis. All experiments were performed in [triplicate](file:///D:/Apps/youdaocidian/Dict/8.9.6.0/resultui/html/index.html#/javascript:;).

**Construction of lentivirus and stable cell lines**

Lentiviral vectors encoding shRNAs were generated using PLKO.1-TRC (Addgene) and designated as LV-shETV1, LV-shPTK2, LV-shc-MET, LV-shELK1, and LV-shControl. LV-shControl is a non-target shRNA control. The vector “pLKO.1-puro Non-Target shRNA Control Plasmid DNA” (purchased from Sigma, SHC016) contains an shRNA insert that does not target any known genes from any species. The shRNA sequences can be found in Supplementary Table S4. Lentiviral vectors encoding human ETV1, PTK2, c-MET, and HGF were constructed in pLV-puro or pLV-neo (Addgene) and designated as LV-ETV1, LV-PTK2, LV-c-MET, and LV-HGF. An empty vector was used as the negative control and was designated as LV-control. The lentivirus and cell infection were produced according to the lentiviral vector protocol recommended by Addgene. Briefly, the lentiviral plasmid and packaging plasmids pMD2. G and psPAX2 (Addgene plasmid #12259 and #12260) were transfected into HEK-293T cells with transfection reagent (Lipofectamine®3000, Thermo Fisher Scientific) and OPTI-MEM media (Invitrogen, MA, USA). The lentiviruses were harvested twice on days 4 and 5. Viruses were filtered with a 0.45-μm filter and stored at -80 °C. For stable cell lines construction, HCC cells were transfected with lentivirus at a multiplicity of infection (MOI) of 10-30 for 12-24 h. 72 h after infection, HCC cells were selected with 2.5 μg/ml puromycin (OriGene) for 2 weeks. The stable cell lines were confirmed by qRT-PCR and western blotting.

**Luciferase reporter assays**

The Dual-Luciferase Reporter Assay (Promega, CA, USA) was used to detect luciferase activity according to the manufacturer’s instructions. In brief, the cells transfected with plasmids were lysed, and the lysates were centrifuged at maximum speed for 1 min. Relative luciferase activity was determined using a ModulusTM TD20/20 Luminometer (Turner Biosystems, CA, USA) and was normalized to Renilla luciferase activity.

**Plasmid construction**

Plasmid construction was performed according to standard procedures. The primers were shown in Supplementary Table S3. For example, the *ETV1* gene complete CDS construct, pCMV-ETV1, was generated by using cDNA from human PBMCs. It was generated with forward and reversed primers incorporating EcoRI and BamHI sites at the 5’ and 3’-ends, respectively. The polymerase chain reaction (PCR) product was cloned into the EcoRI and BamHI sites of the pCMV-Tag2B vector. The *MET* promoter construct (-1699/+257) was generated from human genomic DNA. This construct corresponds to a sequence from -1699 to +257 (relative to the transcriptional start site) of the 5’-flanking region of the human *MET* gene. It was generated with forward and reverse primers incorporating Sac I and Xho I sites at the 5’ and 3’-ends, respectively. The polymerase chain reaction (PCR) product was cloned into the Sac I and XhoI sites of the pGL3-Basic vector (Promega, CA, USA). The 5’-flanking deletion constructs of the *MET* promoter, (-1450/+257) MET, (-826/+257) MET, (-244/+257) MET were similarly generated using the (-1699/+257) MET construct as the template. The ETV1 binding sites in the *MET* promoter were mutated using the QuikChange II Site-Directed Mutagenesis Kit (Stratagene, CA, USA). The constructs were confirmed by DNA sequencing. Other promoter constructs were cloned in the same manner.

**Transient transfection**

The cells were plated at a density of 1 × 10^5^ cells/well in a 24-well plate. After 12-24 h, the cells were co-transfected with 0.6 μg of expression vector plasmids, 0.18 μg of promoter reporter plasmids, and 0.02 μg of pRL-TK plasmids using Lipofectamine 2000 (Invitrogen, MA, USA) according to the manufacturer’s instructions. After 6 h of transfection, the cells were washed and allowed to recover overnight in fresh medium supplemented with 1% FBS for 48 h. Serum-starved cells were used for the assay.

**Chromatin immunoprecipitation Assay (ChIP)**

Cells were immersed in 1% formaldehyde for 10 min at 37 °C to stimulate cross-linking. Then, glycine was used to quench the formaldehyde after cross-linking to stop formaldehyde fixation. After washing with PBS, the cells were resuspended in lysis buffer (1 mM PMSF, 1% SDS, 10 mM EDTA, and 50 mM Tris (pH 8.1) – total volume 300 μl). Sonication was then performed to produce fragmented DNA. A slurry of protein G-Sepharose and herring sperm DNA (Sigma-Aldrich) was used to clear the supernatant. The recovered supernatant was then subjected to a 2-hour incubation period with specific antibodies or an isotype control IgG in the presence of protein G-Sepharose beads and herring sperm DNA, followed by antibody denaturation with 1% SDS in lysis buffer. Precipitated DNA was extracted from the beads by immersing them in a 1.1 M NaHCO_3_ solution and 1% SDS solution at 65 °C for 6 h. Immunoprecipitated DNA was retrieved from the beads by immersion in 1% SDS and a 1.1 M NaHCO_3_ solution at 65 °C for 6 h. The DNA was then purified using a PCR Purification Kit (Qiagen, [Germany](D:/Apps/youdaocidian/Dict/8.9.6.0/resultui/html/index.html#/javascript:;)). The primers were shown in Supplementary Table S3.

For ChIP assays of tissues, cells were first separated from six pairs of fresh frozen HCC tissues and normal liver tissues collected after surgical resection. In detail, surgically extracted tumor tissues were first washed by 1 × cold PBS, 5 min, for three times and added to medium supplemented with antibiotics and antifungal agents. Use a clean razor blade to cut a pie of tissue (around 5 mm^3^) into small piece (typical 1 mm^3^ or smaller). Then, digestion the tissues with DNase I (20 mg/mL; Sigma-Aldrich) and collagenase (1.5 mg/mL; Sigma-Aldrich) and placed on a table concentrator, 37 ℃, for 1 h. At the end of the hour, we filtered the dissociated cells through 70 μm-pore filters rinsed with fresh media. The 1 × red cell lysis was added to the tissues and incubated for 5 min to lysis the red blood cell, followed by another rinse. The dissociated cells were crosslinked using 1% formaldehyde for 10 min at 37 ℃. After cell lysis, the DNA was fragmented by sonication. ChIP grade antibody or IgG (negative control) was used to immunoprecipitate the fragment DNA. Then, qRT-PCR was used to amplify the corresponding binding site on the promoters.

**Cell Counting Kit-8 (CCK8) assay**

For cell proliferation studies, HCC cells were seeded into 96-well plates (5000 cells/well). Six wells of each group were detected every day. The cells were incubated into 100 μl of fresh medium containing 10 μl CCK8 at 37 °C for 2 h, and then the medium was replaced by 100 μl of DMSO and shaken at room temperature for 10 min. The absorbance was measured at 450 nm.

**Colony formation** **assay**

For colony formation assays, HCC cells were seeded into 35 mm dishes (500 cells/dish). Then the cells were incubated at 37 ℃ in 5% CO2 for 2 weeks. Subsequently, the medium was removed. The cells were fixed with 4% paraformaldehyde, stained with 0.1% crystal violet, and imaged with light microscope (Olympus, Japan). Only positive colonies (diameter > 40 um) in the dishes were counted and compared.

***In vivo* tumor growth in the xenograft model**

All animal experiments were approved by the Committee on the Tongji Hospital of Tongji Medical College, Huazhong University of Science and Technology. BALB/C nude mice (male, five weeks old) were housed and cared according to the institutional guidelines for animal care. For the *in vivo* growth assay, suspended treated cells were subcutaneously injected into the flank of each mouse (five mice per group, 1 × 10^6^ cells in 150 μl of PBS per mouse). The mice were weighed and the tumor size was measured using vernier calipers. The tumor volume was calculated using the following equation: V (mm^3^) = 0.5 × L (mm) × W^2^ (mm^2^). After four weeks, all mice were sacrificed. Then, tumor weight was measured. The tumors were then embedded in paraffin and prepared for H&E staining.

**Supplementary Figures**

**Figure S1.**

**

**

**Figure S1.**

1. *ETV1* expression in LIHC and correlation of *ETV1* expression with overall survival were analyzed in LIHC according to the data of The Cancer Genome Atlas (TCGA).

(B-C) RT-qPCR and western blotting analysis of ETV1 expression in normal liver tissues and HCC cell lines.

(D) Cell Counting Kit-8 (CCK8) assay assessing the cell proliferation of the ETV1-overexpressing PLC/PRF/5 cells and ETV1-knockdown MHCC97H cells.

(E) Colony formation assay showing the proliferation of the indicated HCC cells. The representative photos were shown and the cell numbers were quantified.

(F-H) Tumor growth of the indicated HCC cells was assessed by subcutaneous xenograft tumor models. The tumor volume and weight were shown in (F) and (G), the representative images of Ki67 were shown in (H). n = 5 in each group.

(I) The correlation between ETV1 expression and PTK2 or MET expression in TCGA-LIHC and GEO database.

*p < 0.05, ****p < 0.0001. Data were shown as Mean ± SD.

**Figure S2.**

**
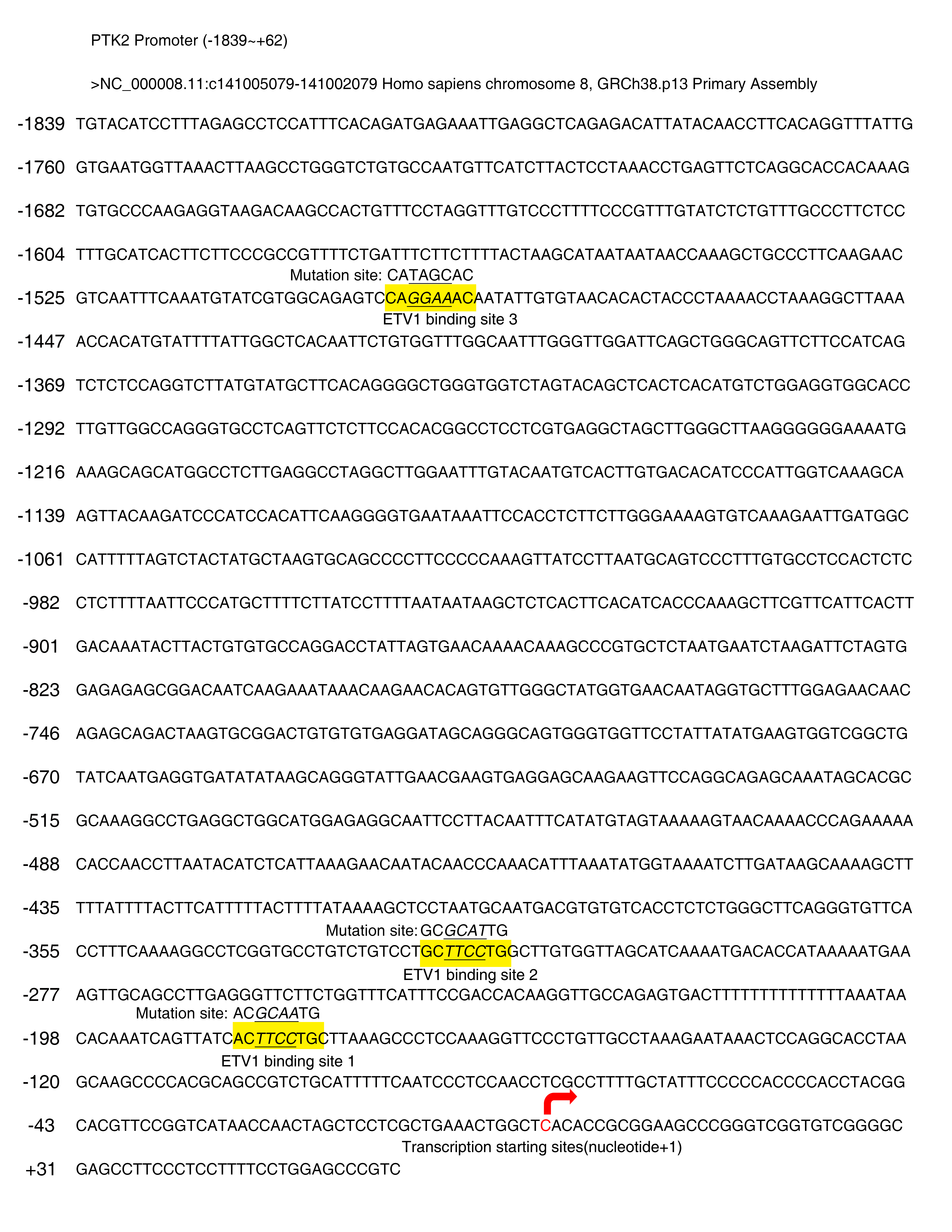
**

**Figure S2. ETV1 binding sites within the promoter regions of *PTK2*.**

The sequences highlighted in yellow represent the three binding sites of ETV1 on the PTK2 promoter, and the arrow represents the transcription initiation sites. The mutagenesis of the promoter sequence were annotated.

**Figure S3.**


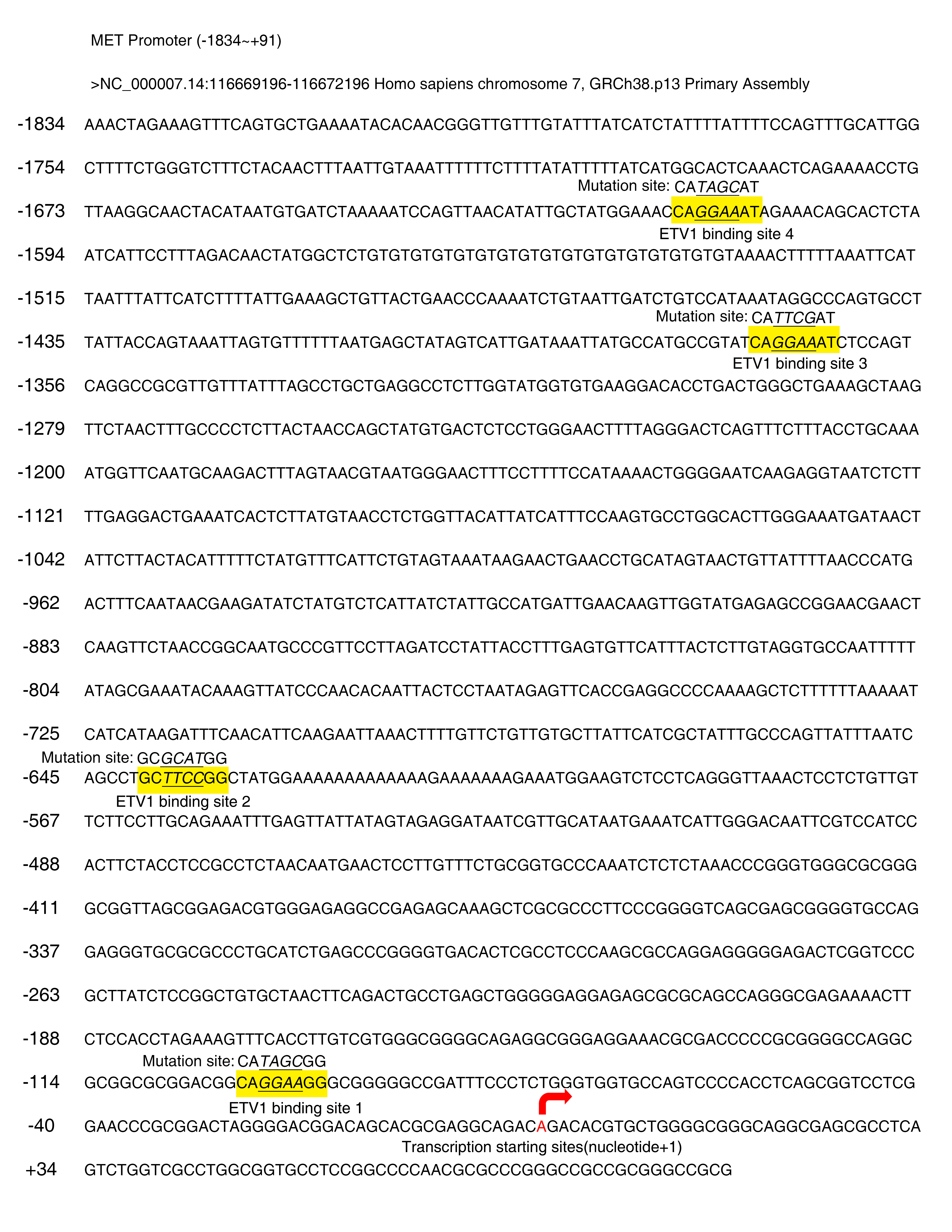


**Figure S3. ETV1 binding sites within the promoter regions of *MET*.**

The sequences highlighted in yellow represent the four binding sites of ETV1 on the *MET* promoter, and the arrow represents the transcription initiation sites. The mutagenesis of the promoter sequence were annotated.

**Figure S4.**


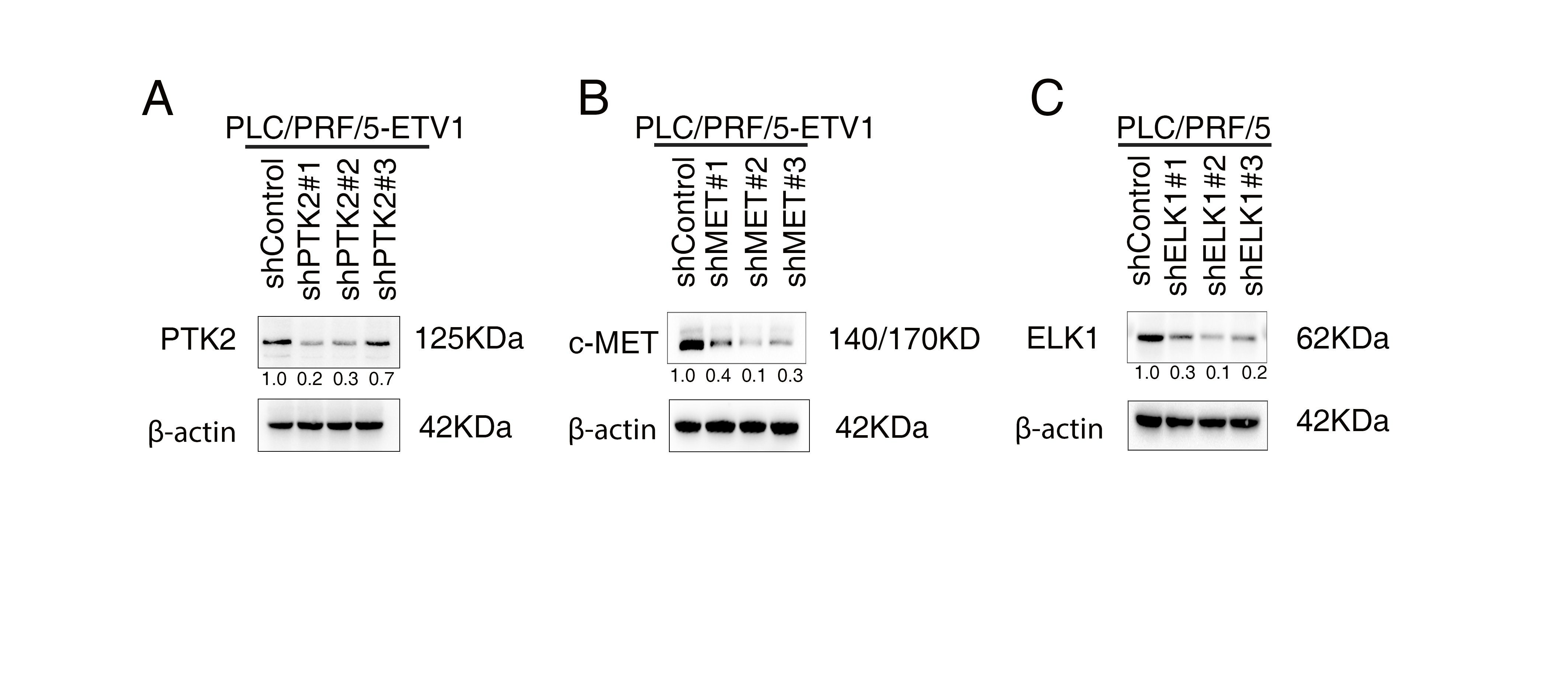


**Figure S4.**

1. C) Western blot verifying PTK2 and MET knockdown effect in PLC/PRF/5-ETV1 cells and ELK1 knockdown effect in PLC/PRF/5 cells.

**Figure S5.**

**
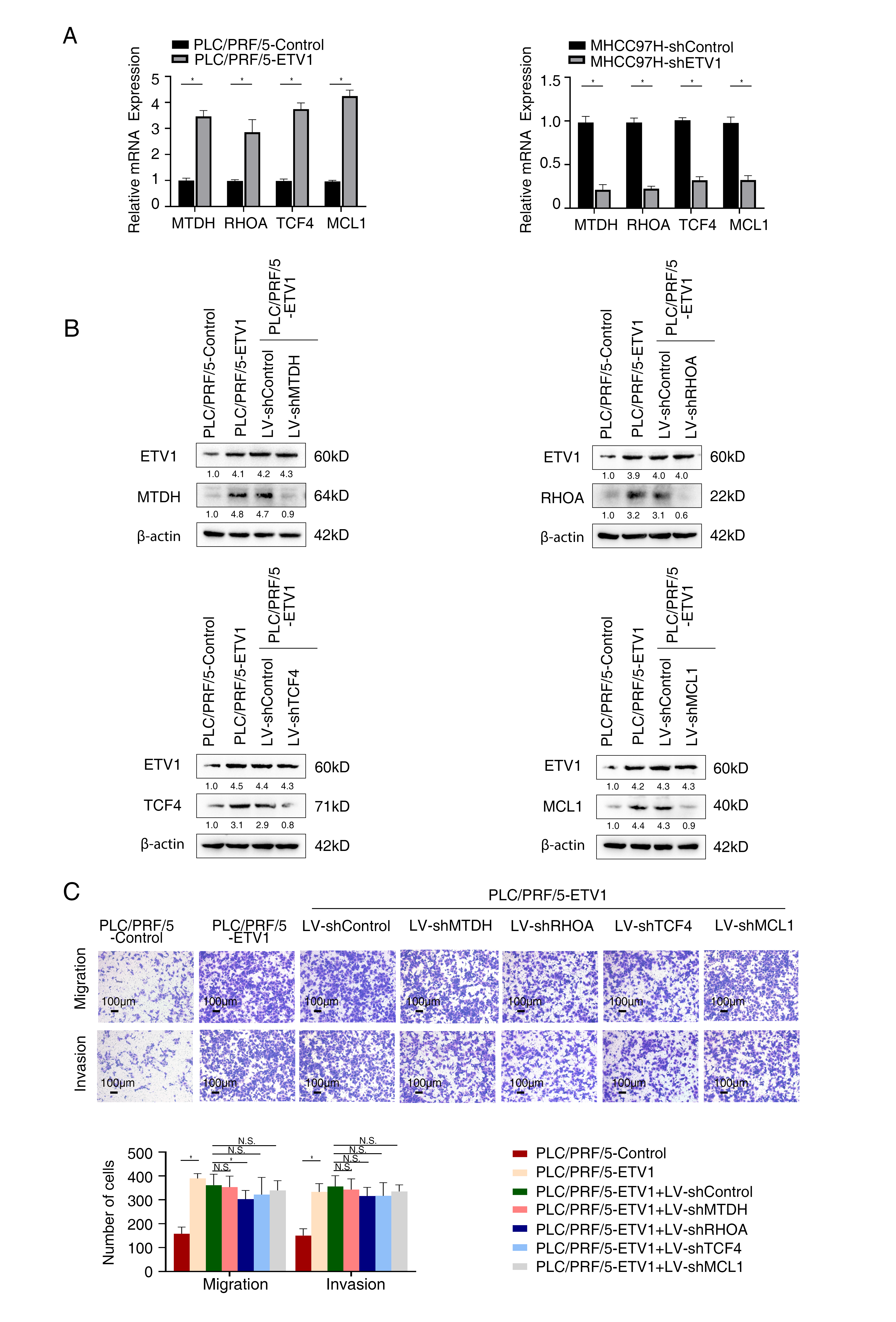
**

**Figure S5.**

(A) The expression levels of MTDH, RHOA, TCF4 and MCL1 were determined in the indicated cells by real-time PCR.

(B) Western blotting assays of MTDH, RHOA, TCF4 and MCL1 in the indicated cells transfected with lentivirus.

(C) The migrating and invasive capability of the indicated cells was determined via transwell assay.

**Figure S6.**

**
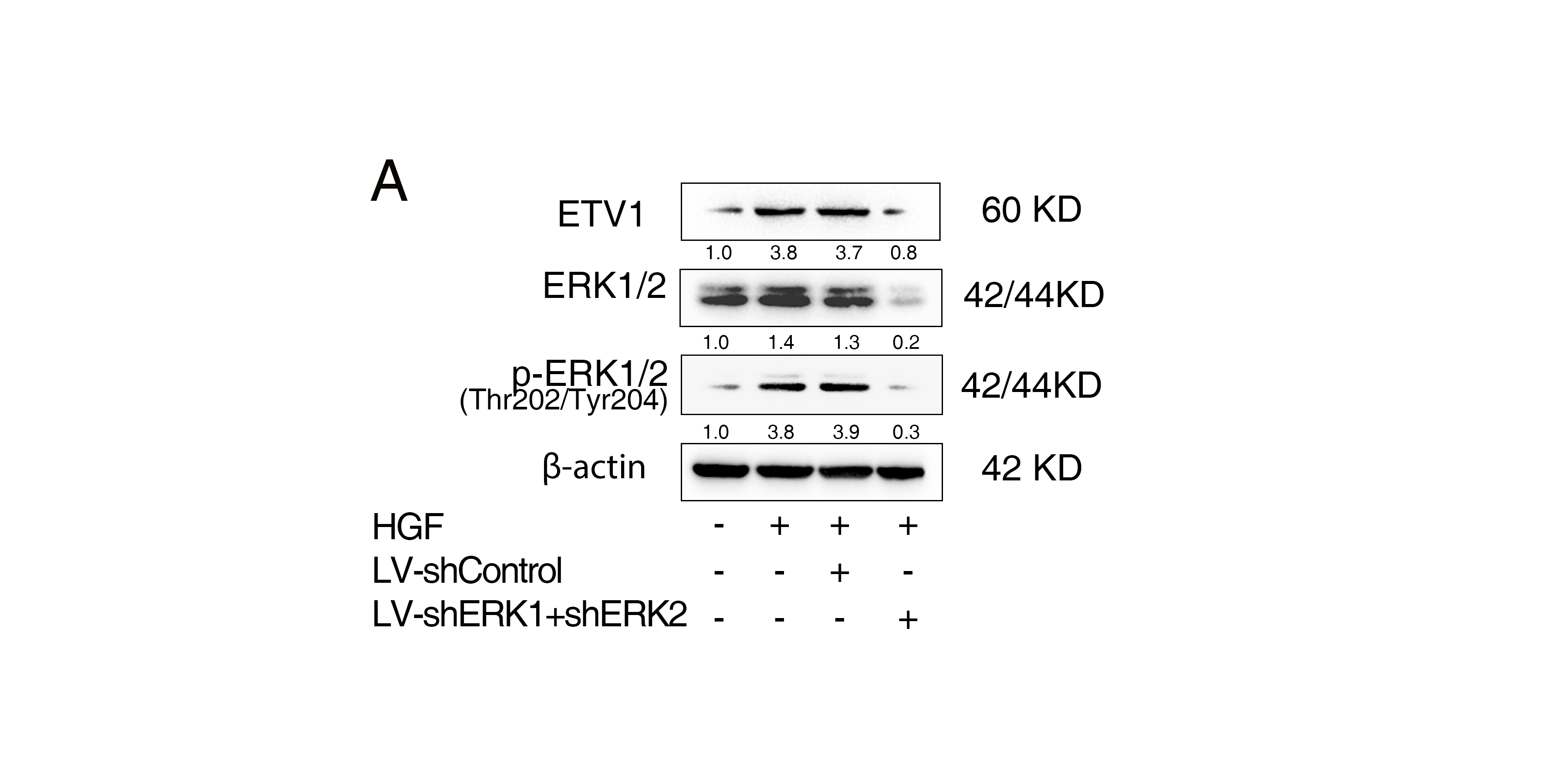
**

**Figure S6.**

(A)The level of ETV1 in the PLC/PRF/5 cells upon HGF treatment with/without ERK1/2 knockdown.

**Figure S7.**


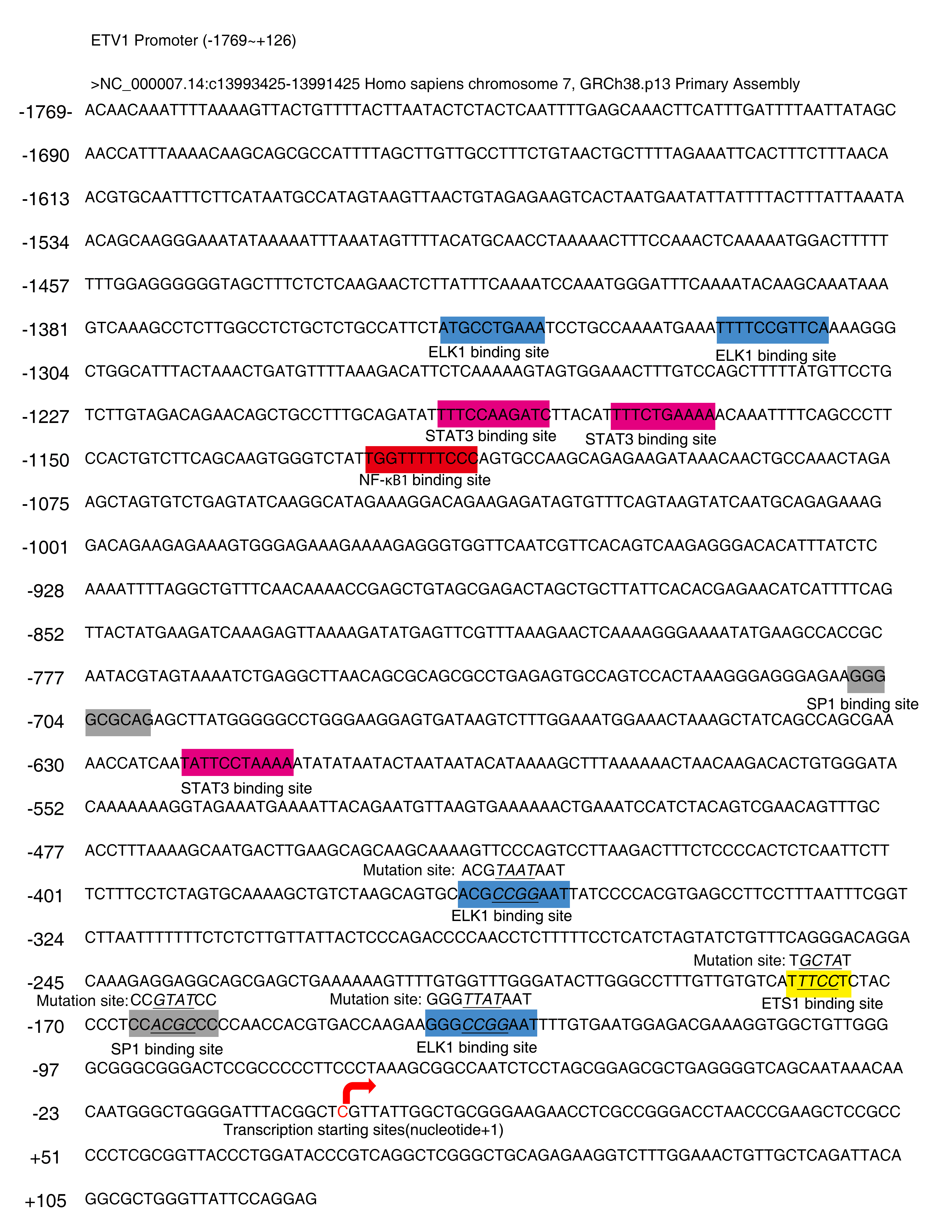


**Figure S7. Transcription factors binding sites within the promoter regions of *ETV1*.**

The sequences highlighted in blue represent the four binding sites of ELK1 on the *ETV1* promoter. The yellow highlighted sequences represent the one binding site of ETS1 on the *ETV1* promoter. The sequences highlighted in grey represent the binding site of SP1 on the *ETV1* promoter. The red highlighted sequences represent the binding site of NF-ΚB1 on the *ETV1* promoter. The pink highlighted sequences represent the binding sequence of STAT3 on the *ETV1* promoter. The arrows represent transcription start sites. The mutagenesis of the promoter sequence were annotated.

**Figure S8.**


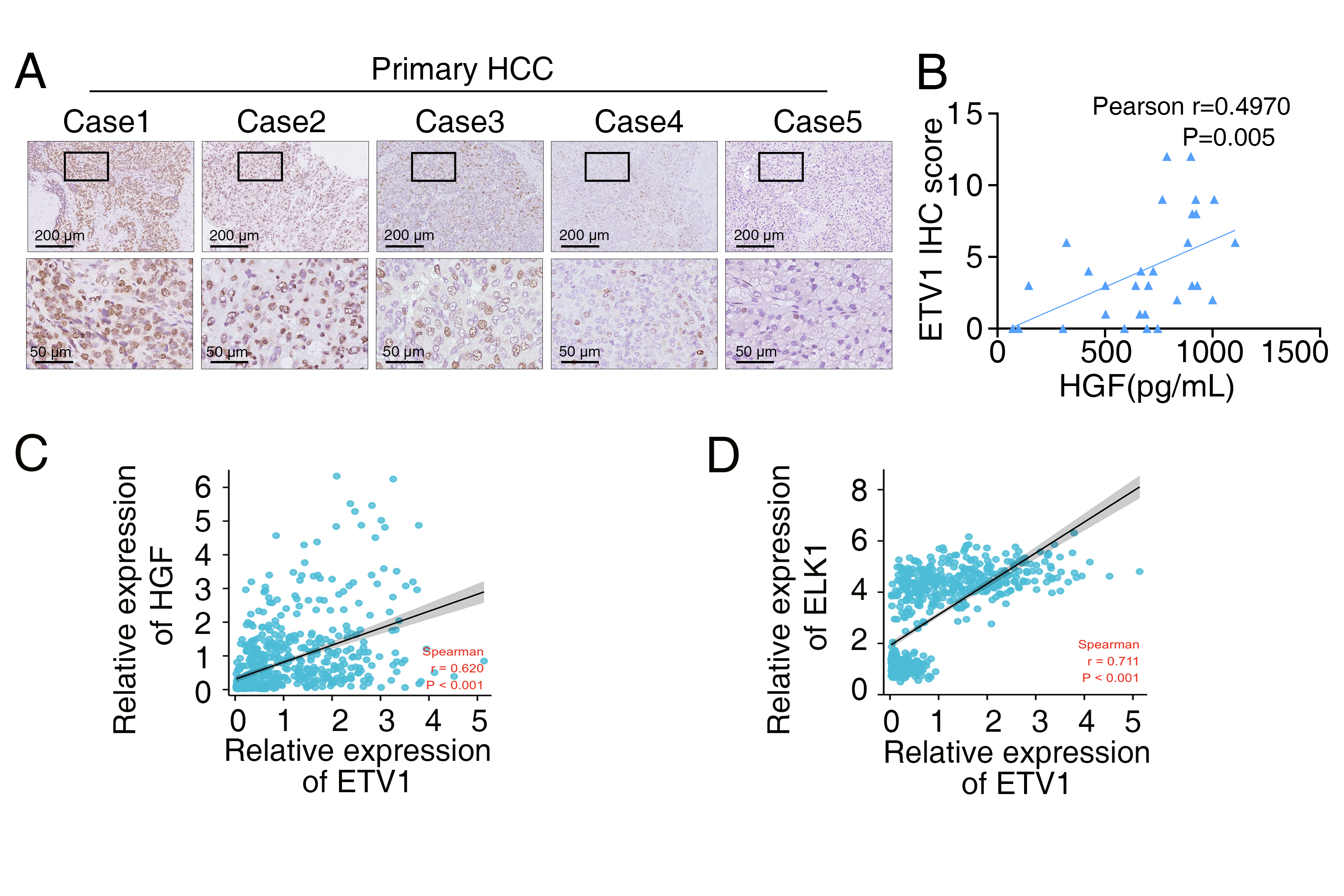


**Figure S8.**

1. Representative IHC staining of ETV1 is shown.
2. Pearson correlation analyses between ETV1 IHC score and the levels of serum HGF in HCC patients. n=30.
3. The correlation between ETV1 expression and HGF expression in TCGA-LIHC and GEO database.
4. The correlation between ETV1 expression and ELK1 expression in TCGA-LIHC and GEO database.

**Figure S9.**





**Figure S9. Effect of ERK1/2 inhibitor on HGF-mediated HCC cell migration and invasion**

(A) Transwell assays displayed the migratory and invasive capacity of the indicated cells upon HGF treatment.

(B) Transwell assays displayed the migratory and invasive capacity of the indicated cells.

**Figure S10.**

**

**

**Figure S10.**

(A) Relative mRNA expression of ETV1, PTK2, and MET in 20 cases of adjacent nontumorous, primary HCC, and metastatic HCC specimens, respectively.

(B-C) Representative IHC staining and IHC scores of ETV1, PTK2, and c-MET in the indicated specimens.

Supplementary Table S1. List of genes differentially expressed in PLC/PRF/5-ETV1 versus PLC/PRF/5-Control cells using a human liver cancer PCR array

| Gene | Fold change | Description |
| --- | --- | --- |
| ITGB1 | 6.11 | Integrin, beta 1 (fibronectin receptor, beta polypeptide, antigen CD29 includes MDF2, MSK12) |
| MET | 6.07 | Met proto-oncogene (hepatocyte growth factor receptor) |
| CCND2 | 5.23 | Cyclin D2 |
| PTK2 | 5.20 | PTK2 protein tyrosine kinase 2 |
| MCL1 | 4.96 | Myeloid cell leukemia sequence 1 (BCL2-related) |
| TCF4 | 3.66 | Transcription factor 4 |
| ADAM17 | 3.56 | ADAM metallopeptidase domain 17 |
| MTDH | 3.44 | Metadherin |
| FADD | 3.37 | Fas (TNFRSF6)-associated via death domain |
| YAP1 | 3.13 | Yes-associated protein 1 |
| PIN1 | 2.85 | Peptidylprolyl cis/trans isomerase, NIMA-interacting 1 |
| RHOA | 2.73 | Ras homolog gene family, member A |
| TLR4 | 2.67 | Toll-like receptor 4 |
| EGFR | 2.45 | Epidermal growth factor receptor |
| FLT1(VEGFR1) | 2.06 | Fms-related tyrosine kinase 1 (vascular endothelial growth factor/vascular permeability factor receptor) |
| CASP8 | 1.49 | Caspase 8, apoptosis-related cysteine peptidase |
| EGF | 1.32 | Epidermal growth factor |
| AKT1 | 1.22 | V-akt murine thymoma viral oncogene homolog 1 |
| IGFBP3 | 1.20 | Insulin-like growth factor binding protein 3 |
| TNFRSF10B | 1.19 | Tumor necrosis factor receptor superfamily, member 10b |
| STAT3 | 1.17 | Signal transducer and activator of transcription 3 (acute-phase response factor) |
| HGF | 1.16 | Hepatocyte growth factor (hepapoietin A; scatter factor) |
| FZD7 | 1.14 | Frizzled family receptor 7 |
| NFKB1 | 1.13 | Nuclear factor of kappa light polypeptide gene enhancer in B-cells 1 |
| CTNNB1(beta-catenin) | 1.10 | Catenin (cadherin-associated protein), beta 1, 88kDa |
| CCL5 | 1.10 | Chemokine (C-C motif) ligand 5 |
| XIAP | 1.08 | X-linked inhibitor of apoptosis |
| BCL2L1 | 1.08 | BCL2-like 1 |
| SMAD7 | 1.07 | SMAD family member 7 |
| BIRC2 | 1.06 | Baculoviral IAP repeat containing 2 |
| EP300 | 1.06 | E1A binding protein p300 |
| SMAD4 | 1.05 | SMAD family member 4 |
| MSH2 | 1.02 | MutS homolog 2, colon cancer, nonpolyposis type 1 (E. coli) |
| VEGFA | 1.02 | Vascular endothelial growth factor A |
| CDKN1B | 1.01 | Cyclin-dependent kinase inhibitor 1B (p27, Kip1) |
| RASSF1 | 1.01 | Ras association (RalGDS/AF-6) domain family member 1 |
| PTEN | 1.00 | Phosphatase and tensin homolog |
| PDGFRA | -1.02 | Platelet-derived growth factor receptor, alpha polypeptide |
| BCL2 | -1.02 | B-cell CLL/lymphoma 2 |
| ANGPT2 | -1.03 | Angiopoietin 2 |
| RB1 | -1.04 | Retinoblastoma 1 |
| BIRC5 | -1.05 | Baculoviral IAP repeat containing 5 |
| LEF1 | -1.09 | Lymphoid enhancer-binding factor 1 |
| IRS1 | -1.09 | Insulin receptor substrate 1 |
| RAC1 | -1.11 | Ras-related C3 botulinum toxin substrate 1 (rho family, small GTP binding protein Rac1) |
| MSH3 | -1.12 | MutS homolog 3 (E. coli) |
| TGFA | -1.14 | Transforming growth factor, alpha |
| CCND1 | -1.15 | Cyclin D1 |
| NRAS | -1.16 | Neuroblastoma RAS viral (v-ras) oncogene homolog |
| TGFB1 | -1.16 | Transforming growth factor, beta 1 |
| FHIT | -1.20 | Fragile histidine triad gene |
| TERT | -1.23 | Telomerase reverse transcriptase |
| SFRP2 | -1.25 | Secreted frizzled-related protein 2 |
| RUNX3 | -1.28 | Runt-related transcription factor 3 |
| SOCS3 | -1.29 | Suppressor of cytokine signaling 3 |
| HHIP | -1.32 | Hedgehog interacting protein |
| IGFBP1 | -1.34 | Insulin-like growth factor binding protein 1 |
| PTGS2 | -1.39 | Prostaglandin-endoperoxide synthase 2 (prostaglandin G/H synthase and cyclooxygenase) |
| CDKN2A | -1.45 | Cyclin-dependent kinase inhibitor 2A (melanoma, p16, inhibits CDK4) |
| GSTP1 | -1.45 | Glutathione S-transferase pi 1 |
| MYC | -1.46 | V-myc myelocytomatosis viral oncogene homolog (avian) |
| BAX | -1.52 | BCL2-associated X protein |
| TNFSF10 | -1.53 | Tumor necrosis factor (ligand) superfamily, member 10 |
| RELN | -1.55 | Reelin |
| TP53 | -1.57 | Tumor protein p53 |
| IGF2 | -1.63 | Insulin-like growth factor 2 (somatomedin A) |
| CDH13 | -1.63 | Cadherin 13, H-cadherin (heart) |
| GADD45B | -1.65 | Growth arrest and DNA-damage-inducible, beta |
| E2F1 | -1.66 | E2F transcription factor 1 |
| CXCR4 | -1.69 | Chemokine (C-X-C motif) receptor 4 |
| PYCARD | -1.71 | PYD and CARD domain containing |
| OPCML | -1.72 | Opioid binding protein/cell adhesion molecule-like |
| TGFBR2 | -2.03 | Transforming growth factor, beta receptor II (70/80kDa) |
| FAS | -2.22 | Fas (TNF receptor superfamily, member 6) |
| HRAS | -2.26 | V-Ha-ras Harvey rat sarcoma viral oncogene homolog |
| CDH1 | -2.31 | Cadherin 1, type 1, E-cadherin (epithelial) |
| CFLAR | -2.35 | CASP8 and FADD-like apoptosis regulator |
| DLC1 | -2.39 | Deleted in liver cancer 1 |
| DAB2IP | -2.46 | DAB2 interacting protein |
| BID | -2.48 | BH3 interacting domain death agonist |
| CDKN1A | -2.55 | Cyclin-dependent kinase inhibitor 1A (p21, Cip1) |
| WT1 | -2.93 | Wilms tumor 1 |
| KDR(VEGFR) | -3.03 | Kinase insert domain receptor (a type III receptor tyrosine kinase) |
| SOCS1 | -3.38 | Suppressor of cytokine signaling 1 |

Supplementary Table S2. List of genes differentially expressed in MHCC97H-shETV1 versus MHCC97H-shControl cells using a human liver cancer PCR array

| Gene | Fold change | Description |  |  |
| --- | --- | --- | --- | --- |
| PTK2 | -5.96 | PTK2 protein tyrosine kinase 2 |  |  |
| CCL5 | -4.59 | Chemokine (C-C motif) ligand 5 |  |  |
| NFKB1 | -4.57 | Nuclear factor of kappa light polypeptide gene enhancer in B-cells 1 |  |  |
| MET | -4.57 | Met proto-oncogene (hepatocyte growth factor receptor) |  |  |
| MTDH | -4.41 | Metadherin |  |  |
| TGFB1 | -4.31 | Transforming growth factor, beta 1 |  |  |
| RHOA | -3.95 | Ras homolog gene family, member A |  |  |
| TCF4 | -3.61 | Transcription factor 4 |  |  |
| RAC1 | -3.27 | Ras-related C3 botulinum toxin substrate 1 (rho family, small GTP binding protein Rac1) |  |  |
| MCL1 | -2.92 | Myeloid cell leukemia sequence 1 (BCL2-related) |  |  |
| EGF | -2.67 | Epidermal growth factor |  |  |
| MSH2 | -1.84 | MutS homolog 2, colon cancer, nonpolyposis type 1 (E. coli) |  |  |
| STAT3 | -1.82 | Signal transducer and activator of transcription 3 (acute-phase response factor) |  |  |
| AKT1 | -1.76 | V-akt murine thymoma viral oncogene homolog 1 |  |  |
| BCL2L1 | -1.63 | BCL2-like 1 |  |  |
| EP300 | -1.64 | E1A binding protein p300 |  |  |
| NRAS | -1.60 | Neuroblastoma RAS viral (v-ras) oncogene homolog |  |  |
| FAS | -1.51 | Fas (TNF receptor superfamily, member 6) |  |  |
| ITGB1 | -1.50 | Integrin, beta 1 (fibronectin receptor, beta polypeptide, antigen CD29 includes MDF2, MSK12) |  |  |
| TLR4 | -1.49 | Toll-like receptor 4 |  |  |
| XIAP | -1.44 | X-linked inhibitor of apoptosis |  |  |
| CTNNB1(beta-catenin) | -1.41 | Catenin (cadherin-associated protein), beta 1, 88kDa |  |  |
| YAP1 | -1.40 | Yes-associated protein 1 |  |  |
| ADAM17 | -1.40 | ADAM metallopeptidase domain 17 |  |  |
| RB1 | -1.39 | Retinoblastoma 1 |  |  |
| CASP8 | -1.36 | Caspase 8, apoptosis-related cysteine peptidase |  |  |
| BIRC2 | -1.33 | Baculoviral IAP repeat containing 2 |  |  |
| CDKN1B | -1.33 | Cyclin-dependent kinase inhibitor 1B (p27, Kip1) |  |  |
| FADD | -1.19 | Fas (TNFRSF6)-associated via death domain |  |  |
| BCL2 | -1.17 | B-cell CLL/lymphoma 2 |  |  |
| SMAD7 | -1.16 | SMAD family member 7 |  |  |
| SFRP2 | -1.14 | Secreted frizzled-related protein 2 |  |  |
| VEGFA | -1.14 | Vascular endothelial growth factor A |  |  |
| IGFBP3 | -1.12 | Insulin-like growth factor binding protein 3 |  |  |
| FLT1(VEGFR1) | -1.11 | Fms-related tyrosine kinase 1 (vascular endothelial growth factor/vascular permeability factor receptor) |  |  |
| SMAD4 | -1.10 | SMAD family member 4 |  |  |
| SOCS3 | -1.08 | Suppressor of cytokine signaling 3 |  |  |
| FZD7 | -1.08 | Frizzled family receptor 7 |  |  |
| ANGPT2 | -1.06 | Angiopoietin 2 |  |  |
| TNFRSF10B | -1.05 | Tumor necrosis factor receptor superfamily, member 10b |  |  |
| CCND2 | -1.04 | Cyclin D2 |  |  |
| MSH3 | -1.01 | MutS homolog 3 (E. coli) |  |  |
| HHIP | 1.14 | Hedgehog interacting protein |  |  |
| RASSF1 | 1.15 | Ras association (RalGDS/AF-6) domain family member 1 |  |  |
| PTEN | 1.16 | Phosphatase and tensin homolog |  |  |
| RUNX3 | 1.17 | Runt-related transcription factor 3 |  |  |
| BIRC5 | 1.17 | Baculoviral IAP repeat containing 5 |  |  |
| PDGFRA | 1.19 | Platelet-derived growth factor receptor, alpha polypeptide |  |  |
| CDKN2A | 1.20 | Cyclin-dependent kinase inhibitor 2A (melanoma, p16, inhibits CDK4) |  |  |
| RELN | 1.24 | Reelin |  |  |
| CXCR4 | 1.31 | Chemokine (C-X-C motif) receptor 4 |  |  |
| HGF | 1.36 | Hepatocyte growth factor (hepapoietin A; scatter factor) |  |  |
| IRS1 | 1.41 | Insulin receptor substrate 1 |  |  |
| EGFR | 1.45 | Epidermal growth factor receptor |  |  |
| FHIT | 1.45 | Fragile histidine triad gene |  |  |
| MYC | 1.46 | V-myc myelocytomatosis viral oncogene homolog (avian) |  |  |
| CDH1 | 1.49 | Cadherin 1, type 1, E-cadherin (epithelial) |  |  |
| HRAS | 1.51 | V-Ha-ras Harvey rat sarcoma viral oncogene homolog |  |  |
| PYCARD | 1.57 | PYD and CARD domain containing |  |  |
| OPCML | 1.62 | Opioid binding protein/cell adhesion molecule-like |  |  |
| KDR(VEGFR) | 1.64 | Kinase insert domain receptor (a type III receptor tyrosine kinase) |  |  |
| CDKN1A | 1.70 | Cyclin-dependent kinase inhibitor 1A (p21, Cip1) |  |  |
| WT1 | 1.75 | Wilms tumor 1 |  |  |
| CCND1 | 1.91 | Cyclin D1 |  |  |
| PIN1 | 1.92 | Peptidylprolyl cis/trans isomerase, NIMA-interacting 1 |  |  |
| PTGS2 | 1.95 | Prostaglandin-endoperoxide synthase 2 (prostaglandin G/H synthase andcyclooxygenase) |  |  |
| TGFBR2 | 2.01 | Transforming growth factor, beta receptor II (70/80kDa) |  |  |
| TP53 | 2.07 | Tumor protein p53 |  |  |
| TGFA | 2.12 | Transforming growth factor, alpha |  |  |
| BAX | 2.34 | BCL2-associated X protein |  |  |
| TERT | 2.51 | Telomerase reverse transcriptase |  |  |
| TNFSF10 | 2.56 | Tumor necrosis factor (ligand) superfamily, member 10 |  |  |
| BID | 2.70 | BH3 interacting domain death agonist |  |  |
| GADD45B | 3.45 | Growth arrest and DNA-damage-inducible, beta |  |  |
| IGFBP1 | 3.47 | Insulin-like growth factor binding protein 1 |  |  |
| IGF2 | 3.69 | Insulin-like growth factor 2 (somatomedin A) |  |  |
| CDH13 | 3.80 | Cadherin 13, H-cadherin (heart) |  |  |
| DLC1 | 4.00 | Deleted in liver cancer 1 |  |  |
| DAB2IP | 4.01 | DAB2 interacting protein |  |  |
| GSTP1 | 4.05 | Glutathione S-transferase pi 1 |  |  |
| SOCS1 | 4.15 | Suppressor of cytokine signaling 1 |  |  |
| E2F1 | 4.25 | E2F transcription factor 1 |  |  |
| LEF1 | 4.26 | Lymphoid enhancer-binding factor 1 |  |  |
| CFLAR | 4.45 | CASP8 and FADD-like apoptosis regulator |  |  |

Supplementary Table S3. Primer sequences used in the study

| **Primer name** | **Primer sequences** | **Enzyme** |
| --- | --- | --- |
| **Primers for real-time PCR:** |  |  |
| ETV1 sense | 5’-TGGCAGTTTTTGGTAGCTCTTC-3’ |  |
| ETV1 antisense | 5’-CGGAGTGAACGGCTAAGTTTATC-3’ |  |
| MET sense: | 5’-AGCGTCAACAGAGGGACCT-3’ |  |
| MET antisense: | 5’-GCAGTGAACCTCCGACTGTATG-3’ |  |
| PTK2 sense: | 5’-TGGTGCAATGGAGCGAGTATT-3’ |  |
| PTK2 antisense: | 5’-CAGTGAACCTCCTCTGACCG-3’ |  |
| β-actin sense: | 5’-CATGTACGTTGCTATCCAGGC-3’ |  |
| β-actin antisense: | 5’-CTCCTTAATGTCACGCACGAT-3’ |  |
| **Primers for MET promoter construct:** | |  |
| (-1699/+257) MET sense: | 5’-TATAGAGCTCCATGGCACTCAAACTCAG-3’ | Sac I |
| (-1450/+257) MET sense: | 5’-TATAGAGCTCATAGGCCCAGTGCCTTAT-3’ | Sac I |
| (-826/+257) MET sense: | 5’-TATAGAGCTCACTCTTGTAGGTGCCAAT-3’ | Sac I |
| (-244/+257) MET sense: | 5’-TATAGAGCTCAACTTCAGACTGCCTGAG-3’ | Sac I |
| (-54/+257) MET sense: | 5’-TATAGAGCTCCTCAGCGGTCCTCGGAAC-3’ | Sac I |
| Antisense: | 5’-ATATCTCGAGCTCCGCATCTGCTCACAA-3’ | Xho I |
| **Primers for MET promoter site-directed mutagenesis:**  ETV1 binding site: | |  |
| binding site 4 mutation sense: | 5’-CTATGGAAACCAtagcATAGAAACAG  CA-3’ |  |
| binding site 4 mutation antisense: | 1. TGCTGTTTCTATgctaTGGTTTCCATAG   -3’ | |
| binding site 3 mutation sense: | 5’-CATGCCGTATCAttcgATCTCCAGTCA  G-3’ | |
| binding site 3 mutation antisense: | 5’-CTGACTGGAGATcgaaTGATACGGCATG-3’ |  |
| binding site 2 mutation sense: | 5’-TAATCAGCCTGCgcatGGCTATGGAA  AA-3’ |  |
| binding site 2 mutation antisense: | 5’-CGGCCCCCGCCCgctaTGCCGTCCGC  GC-3’ |  |
| binding site 1 mutation sense: | 5’-GCGCGGACGGCAtagcGGGCGGGGG  CCG-3’ |  |
| binding site 1 mutation antisense: | 5’-CGGCCCCCGCCCgctaTGCCGTCCGC  GC-3’ |  |
| **Primers used for ChIP in the MET promoter:** | |  |
| distant region sense: | 5’-CTGTACCTCAGCAATGTC-3’ |  |
| distant region antisense: | 5’-GCTAAGCCAGCATCTGTT-3’ |  |
| binding site 1 sense: | 5’-TGTGCTAACTTCAGACTG-3’ |  |
| binding site 1 antisense: | 5’-GGTTCCGAGGACCGCTGA-3’ |  |
| **Primers for PTK2 promoter construct:** | |  |
| (-1670/+153) PTK2 sense: | 5’TATAGAGCTCGTAAGACAAGCCACTGTT-3’ | Sac I |
| (-575/+153) PTK2 sense: | 5’TATAGAGCTCCATGGAGAGGCAATTCCT-3’ | Sac I |
| (-43/+153) PTK2 sense: | 5’TATAGAGCTCCACGTTCCGGTCATAACC--3’ | Sac I |
| Antisense: | 5’ATATCTCGAGACTTAGAAGTCCACTGGA-3’ | Xho I |
| **Primers for PTK2 promoter site-directed mutagenesis:**  ETV1 binding site: | |  |
| binding site 3 mutation sense: | 5’-TGGCAGAGTCCAtagcACAATATTGTGT-3’ |  |
| binding site 3 mutation antisense: | 5’-ACACAATATTGTgctaTGGACTCTGCCA-3’ |  |
| binding site 2 mutation sense: | 5’-TGTCTGTCCTGCgcatTGGCTTGTGGTT-3’ |  |
| binding site 2 mutation antisense: | 5’-AACCACAAGCCAatgcGCAGGACAGACA  -3’ | |
| binding site 1 mutation sense: | 5’-ATCAGTTATCACgcaaTGCTTAAAGCCC  -3’ | |
| binding site 1 mutation antisense: | 5’-GGGCTTTAAGCAttgcGTGATAACTGAT-3’ |  |
| **Primers used for ChIP in the PTK2 promoter:** | |  |
| distant region sense: | 5’-GCAATGCTATAGTCGATC-3’ |  |
| distant region antisense: | 5’-ATTGAGACCATCCTGGCT-3’ |  |
| binding site 1/2 sense: | 5’-AATGACGTGTGTCACCTC-3’ |  |
| binding site 1/2 antisense: | 5’-AGTTTCAGCGAGGAGCTA-3’ |  |
| **Primers for ETV1 promoter construct:** | |  |
| (-1580/+129) ETV1 sense: | 5’-TATAGAGCTCAACTGTAGAGAAGTCACT-3’ | Sac I |
| (-892/+129) ETV1 sense: | 5’-TATAGAGCTCCGAGACTAGCTGCTTATT-3’ | Sac I |
| (-463/+129) ETV1 sense: | 5’-TATAGAGCTCATCTAGTATCTGTTTCAG-3’ | Sac I |
| (-70/+129) ETV1 sense: | 5’-TATAGAGCTCAAGCGGCCAATCTCCTAG-3’ | Sac I |
| antisense: | 5’-ATATCTCGAGGCCTGTAATCTGAGCAAC’-3’ | Xho I |
| **Primers for ETV1 promoter site-directed mutagenesis:** | |  |
| binding site 4 mutation sense: | 5’-TAAGCAGTGCACGtaatAATTATCCCCACG-3’ |  |
| binding site 4 mutation antisense: | 5’-GCTGGGGATAATTattaGCTGCACTGCTTA-3’ |  |
| binding site 3 mutation sense: | 5’-TGTTGTGTCATgctaTCTACCCCTCC-3’ |  |
| binding site 3 mutation antisense: | 5’-GGAGGGGTAGAtagcATGACACAACA-3’ |  |
| binding site 2 mutation sense: | 5’-CTCTACCCCTCCgtatCCCCAACCACGT-3’ |  |
| binding site 2 mutation antisense: | 5’-ACGTGGTTGGGGatacGGAGGGGTAGAG-3’ |  |
| binding site 1 mutation sense: | 5’-TGACCAAGAAGGGttatAATTTTGTGAATG-3’ |  |
| binding site 1 mutation antisense: | 5’-CATTCACAAAATTataaCCCTTCTTGGTCA-3’ |  |
| **Primers used for ChIP in the ETV1 promoter:** | |  |
| distant region sense: | 5’-CAATTTTGAGCAAACTTC-3’ |  |
| distant region antisense: | 5’-AGTGACTTCTCTACAGTT-3’ |  |
| binding site 1 sense: | 5’-ATCTAGTATCTGTTTCAG-3’ |  |
| binding site 1 antisense: | 5’-CTAGGAGATTGGCCGCTT-3’ |  |

Supplementary Table S4. Knockdown shRNA sequences used in this study

| Gene | Sequence |
| --- | --- |
| ETV1 |  |
| shRNA-1 | GTGGGAGTAATCTAAACATTT |
| shRNA-2 | CCACAGTCCATGTTCAGAAAT |
| shRNA-3 | CGACCCAGTGTATGAACACAA |
| MET |  |
| shRNA-1 | CACTGCTTTAATAGGACACTT |
| shRNA-2 | CAGAATGTCATTCTACATGAG |
| shRNA-3 | CATCAGAACCAGAGGCTTGGT |
| PTK2 |  |
| shRNA-1 | GATGTTGGTTTAAAGCGATTT |
| shRNA-2 | CCGATTGGAAACCAACATATA |
| shRNA-3 | CAACAGGTGAAGAGCGATTAT |
| ELK1 |  |
| ShRNA-1 | CCCAAGGAAGAGTTGGAAGTT |
| ShRNA-2 | CCTGCTTCCTACGCATACATT |
| ShRNA-3 | CCCAAGAGTAACTCTCATTAT |

|  |  | Cohort I | |  | Cohort II | |  |
| --- | --- | --- | --- | --- | --- | --- | --- |
| Clinicopathological variables | | Tumor PTK2 expression | | *P* Value | Tumor PTK2 expression | | *P* Value |
|  |  | Negative (n=132) | Positive (n=128) |  | Negative (n=135) | Positive (n=145) |  |
| Age |  | 51.87 | 52.06 | 0.292 | 51.12 | 50.88 | 0.471 |
|  |  | （11.981） | （10.580） |  | （10.230） | （9.633） |  |
| Sex | female | 25 | 24 | 1.000 | 21 | 23 | 1.000 |
|  | male | 107 | 104 |  | 114 | 122 |  |
| Serum AFP | ≤20ng/ml | 36 | 31 | 0.671 | 28 | 27 | 0.764 |
|  | >20ng/ml | 96 | 97 |  | 107 | 118 |  |
| Virus infection | HBV | 99 | 93 | 0.852 | 107 | 118 | 0.120 |
|  | HCV | 12 | 13 |  | 10 | 9 |  |
|  | HBV+HCV | 7 | 5 |  | 4 | 11 |  |
|  | none | 14 | 17 |  | 14 | 7 |  |
| Cirrrhosis | absent | 39 | 33 | 0.580 | 37 | 39 | 1.000 |
|  | present | 93 | 95 |  | 98 | 106 |  |
| Child-pugh score | Class A | 101 | 95 | 0.774 | 114 | 119 | 0.634 |
|  | Class B | 31 | 33 |  | 21 | 26 |  |
| Tumor number | single | 83 | 71 | 0.256 | 106 | 104 | 0.215 |
|  | multiple | 49 | 57 |  | 29 | 41 |  |
| Maximal tumor | ≤5cm | 58 | 50 | 0.452 | 94 | 64 | <0.001 |
| size | >5cm | 74 | 78 |  | 41 | 81 |  |
| Tumor | present | 86 | 59 | 0.003 | 108 | 87 | <0.001 |
| encapsulation | absent | 46 | 69 |  | 27 | 58 |  |
| Microvascular | absent | 83 | 59 | 0.009 | 88 | 65 | 0.001 |
| invasion | present | 49 | 69 |  | 47 | 80 |  |
| Tumor | I-II | 113 | 93 | 0.014 | 114 | 95 | <0.001 |
| differentiation | III-Ⅳ | 19 | 35 |  | 21 | 50 |  |
| TNM stage | I-II | 113 | 85 | <0.001 | 120 | 98 | <0.001 |
|  | III | 19 | 43 |  | 15 | 47 |  |

Supplementary Table S5. Correlation between PTK2 expression and clinicopathological characteristics of HCCs in two independent cohorts of human HCC tissues

Supplementary Table S6. Correlation between c-MET expression and clinicopathological characteristics of HCCs in two independent cohorts of human HCC tissues

|  |  | Cohort I | |  | Cohort II | |  |
| --- | --- | --- | --- | --- | --- | --- | --- |
| Clinicopathological variables | | Tumor c-MET expression | | *P* Value | Tumor c-MET expression | | *P* Value |
|  |  | Negative (n=123) | Positive (n=137) |  | Negative (n=125) | Positive (n=155) |  |
| Age |  | 52.83 | 51.19 | 0.789 | 50.66 | 51.26 | 0.162 |
|  |  | （11.301） | （11.268） |  | （9.129） | （10.516） |  |
| Sex | female | 19 | 30 | 0.206 | 17 | 27 | 0.413 |
|  | male | 104 | 107 |  | 108 | 128 |  |
| Serum AFP | ≤20ng/ml | 33 | 34 | 0.777 | 27 | 28 | 0.545 |
|  | >20ng/ml | 90 | 103 |  | 98 | 127 |  |
| Virus infection | HBV | 94 | 98 | 0.663 | 102 | 123 | 0.293 |
|  | HCV | 9 | 16 |  | 11 | 8 |  |
|  | HBV+HCV | 6 | 6 |  | 6 | 9 |  |
|  | none | 14 | 17 |  | 6 | 15 |  |
| Cirrrhosis | absent | 31 | 41 | 0.409 | 35 | 41 | 0.788 |
|  | present | 92 | 96 |  | 90 | 114 |  |
| Child-pugh score | Class A | 96 | 100 | 0.388 | 108 | 125 | 0.260 |
|  | Class B | 27 | 37 |  | 17 | 30 |  |
| Tumor number | single | 82 | 72 | 0.023 | 99 | 111 | 0.166 |
|  | multiple | 41 | 65 |  | 26 | 44 |  |
| Maximal tumor | ≤5cm | 51 | 57 | 1.000 | 87 | 71 | <0.001 |
| size | >5cm | 72 | 80 |  | 38 | 84 |  |
| Tumor | present | 81 | 64 | 0.003 | 104 | 91 | <0.001 |
| encapsulation | absent | 42 | 73 |  | 21 | 64 |  |
| Microvascular | absent | 80 | 62 | 0.002 | 81 | 72 | 0.003 |
| invasion | present | 43 | 75 |  | 44 | 83 |  |
| Tumor | I-II | 110 | 96 | <0.001 | 105 | 104 | 0.001 |
| differentiation | III-Ⅳ | 13 | 41 |  | 20 | 51 |  |
| TNM stage | I-II | 106 | 92 | <0.001 | 110 | 108 | <0.001 |
|  | III | 17 | 45 |  | 15 | 47 |  |
